# Supplementary material for: Simultaneous nanoscale imaging of local conductivity and chemical potential in a quantum Hall isospin ferromagnet
Source: Nat Commun. 2026 Jun 25;17:7986. doi: 10.1038/s41467-026-74915-y (PMC13448896; doi:10.1038/s41467-026-74915-y)
Supplement: Supplementary file 1 [file 41467_2026_74915_MOESM1_ESM.pdf]

Supplementary Information for

Simultaneous nanoscale imaging of local conductivity and chemical potential in a quantum Hall isospin ferromagnet

Jiawei Hu[1,2,#], Shiyu Zhu[1,2,#]✉, Bohao Li[3,#], Yunhao Wang[1,2,#], Shuigang Xu[4], Zhihai Cheng[5], Chengmin Shen[1,2], Andre K. Geim[6], Fengcheng Wu[3]✉, Hong-Jun Gao[1,2]✉

[1]Beijing National Laboratory for Condensed Matter Physics, Institute of Physics, Chinese Academy of Sciences, Beijing 100190, China

[2]School of Physical Sciences, University of Chinese Academy of Sciences, Beijing 100049, China

[3]School of Physics and Technology, Wuhan University, Wuhan 430072, China

[4]Key Laboratory for Quantum Materials of Zhejiang Province, Department of Physics, School of Science, Westlake University, Hangzhou 310030, China

[5]Key Laboratory of Quantum State Construction and Manipulation (Ministry of Education), School of Physics, Renmin University of China, Beijing 100872, China

[6]Department of Physics and Astronomy, University of Manchester, Manchester, UK

[#]These authors contributed equally: Jiawei Hu, Shiyu Zhu, Bohao Li, and Yunhao Wang.

✉ Corresponding author, E -mail: syzhu@iphy.ac.cn; wufcheng@whu.edu.cn; hjgao@iphy.ac.cn.

Supplementary Figure 1

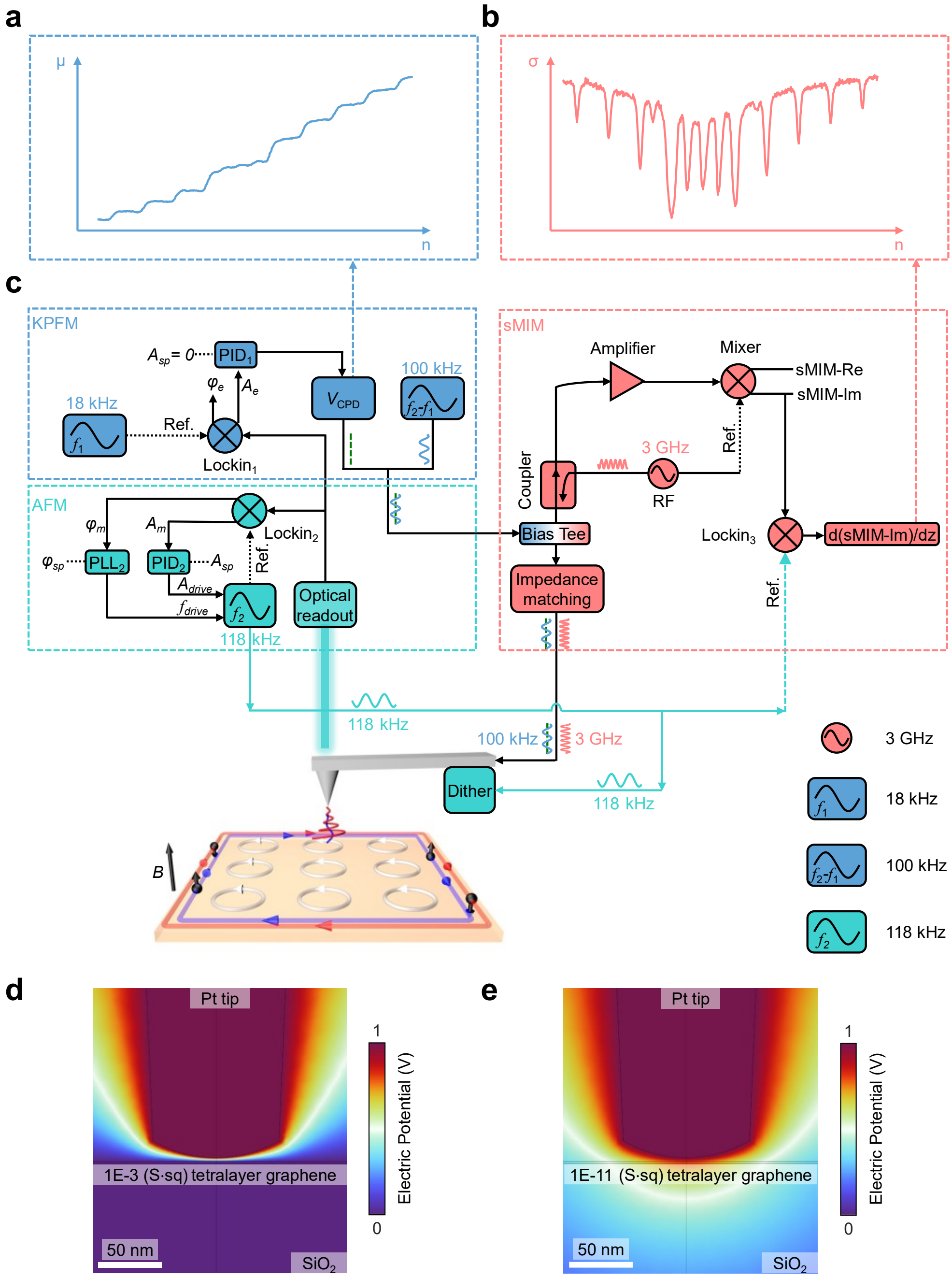

Supplementary Fig. 1 | Working principle of SCCM technique. a, **b**, Schematic of chemical potential μ (**a**) and local conductivity σ (**b**) as a function of carrier density n on BG at $B = 9$ T. These two quantities are extracted by the technique in **c**. **c**, Schematic of SCCM technique, which is divided to three parts, including KPFM, sMIM and AFM. **d**, **e**, Quasi-static field distribution of tip-sample system by finite element simulation. At conductive regime (σ = 1E-3 S·sq), microwave is fully screened by graphene (**d**). At insulating regime (σ = 1E-11 S·sq), microwave penetrates graphene and reflects its electrical properties (**e**). The simulated sMIM response in Fig. 1**b** right is obtained by calculating tip-sample admittance as graphene conductivity variation.

Supplementary Figure 2

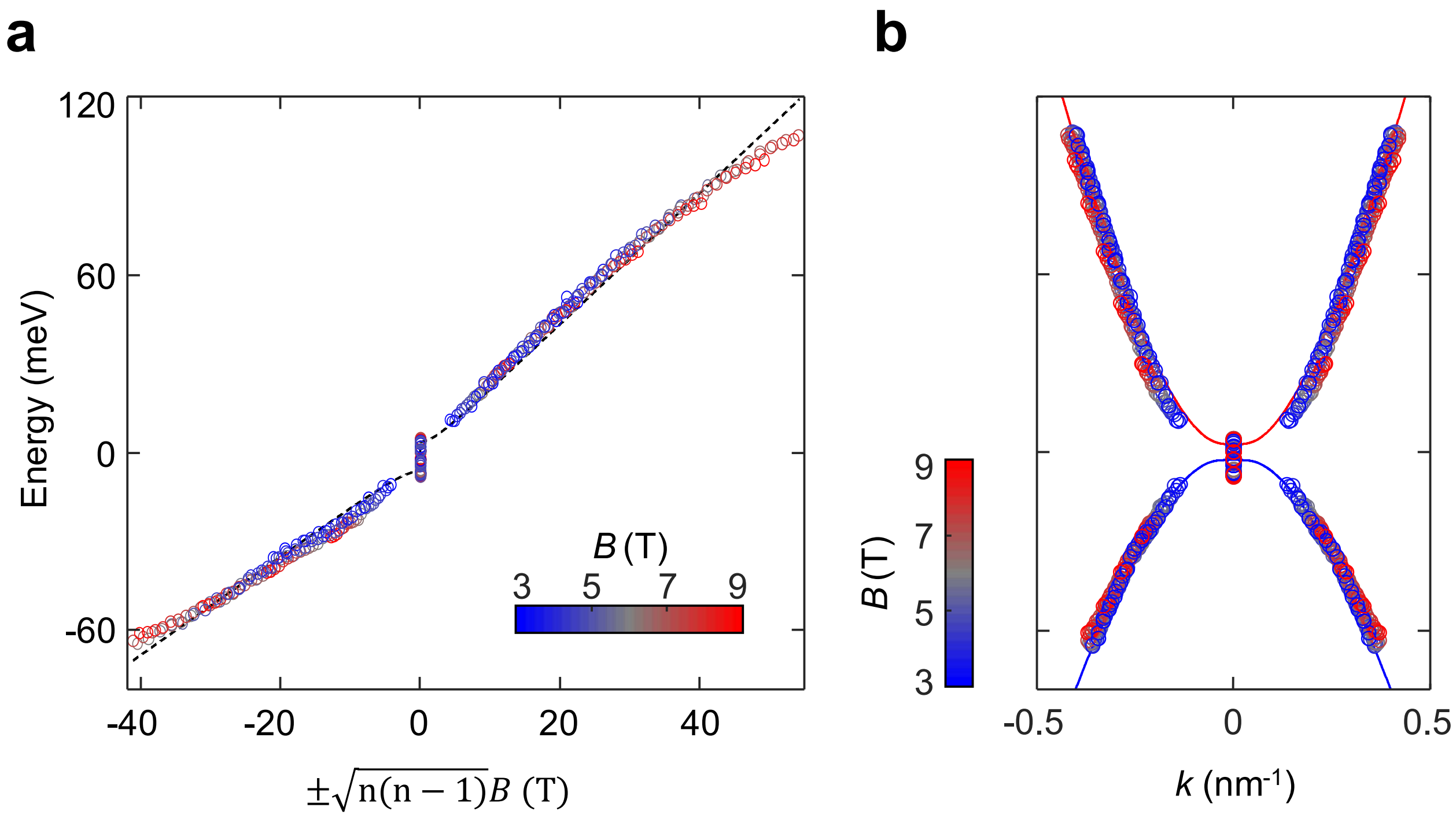

Supplementary Fig. 2 | Landau quantization in BG. a, LL energies plotted against $\pm\sqrt{\mathrm{n}(\mathrm{n}-1)}B$, collapse to a universal curve. The dotted line displays the calculated LL energy using equation (1) with $m_e^* = 0.052\, m_e$ and $m_h^* = 0.069\, m_e$, which matches well with the experimental LL energy curves**. b**, The energy-momentum dispersion obtained from effective band dispersion of the BG. The momentum is determined by the Onsager relation[31] $k_\mathrm{n} = \sqrt{2e\mathrm{n}B/\hbar}$ and the circles are data extracted from **a**. The curves are theoretical fits to the experimental data. We obtain $\gamma_0 = 2.56$ eV, $\gamma_1 = 0.43$ eV, $\gamma_3 = 0.1$ eV, $\gamma_4 = 0.16$ eV, $\delta = 18$ meV, and $u = 5$ meV, which are consistent well with previous results[32]. Calculated effective mass of electron and hole band are $m_e^* = 0.052\, m_e$ and $m_h^* = 0.069\, m_e$, respectively.

Supplementary Figure 3

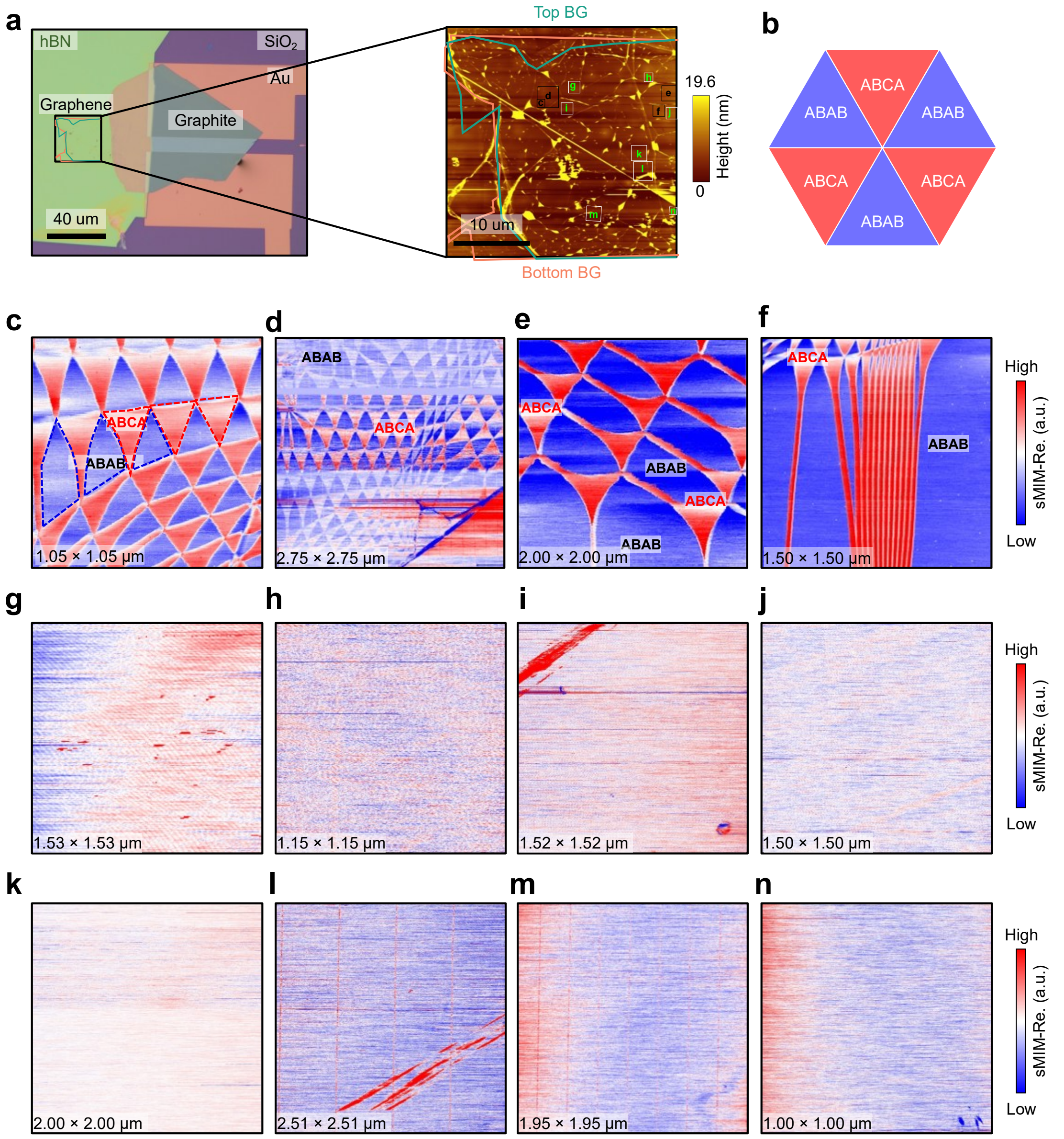

Supplementary Fig. 3 | Optical photo and moiré superlattice of TDBG device. a, Optical photo of device structure (left) and a magnified AFM topography (right) of device at RT, showing several wrinkles confining blocks. **b**, Cartoon of moiré superlattice domain structure of marginally TDBG. **c-f**, Moiré superlattice of TDBG by sMIM-Re mapping at RT. The scan region is 1.05, 2.75, 2.00 and 1.50 μm, respectively. These regions show the convex ABAB and concave ABCA domains in marginally TDBG. The moiré superlattice size varies from ~100 nm to ~500 nm, while the quasi-hexagonal order and ABAB/ABCA configuration maintain. **g-n**, Additional sMIM-Re mappings acquired at RT across various widely separated regions, displaying a complete absence of moiré superlattices and confirming the dominance of uniform ABAB stacking. The moiré region in panel (f) is directly adjacent to the moiré-free region in panel (j), clearly capturing the structural evolution from a mixed ABCA/ABAB moiré state into a pure ABAB phase, with a similar spatial adjacency observed between the regions in panel (d) and panel (i).

Supplementary Figure 4

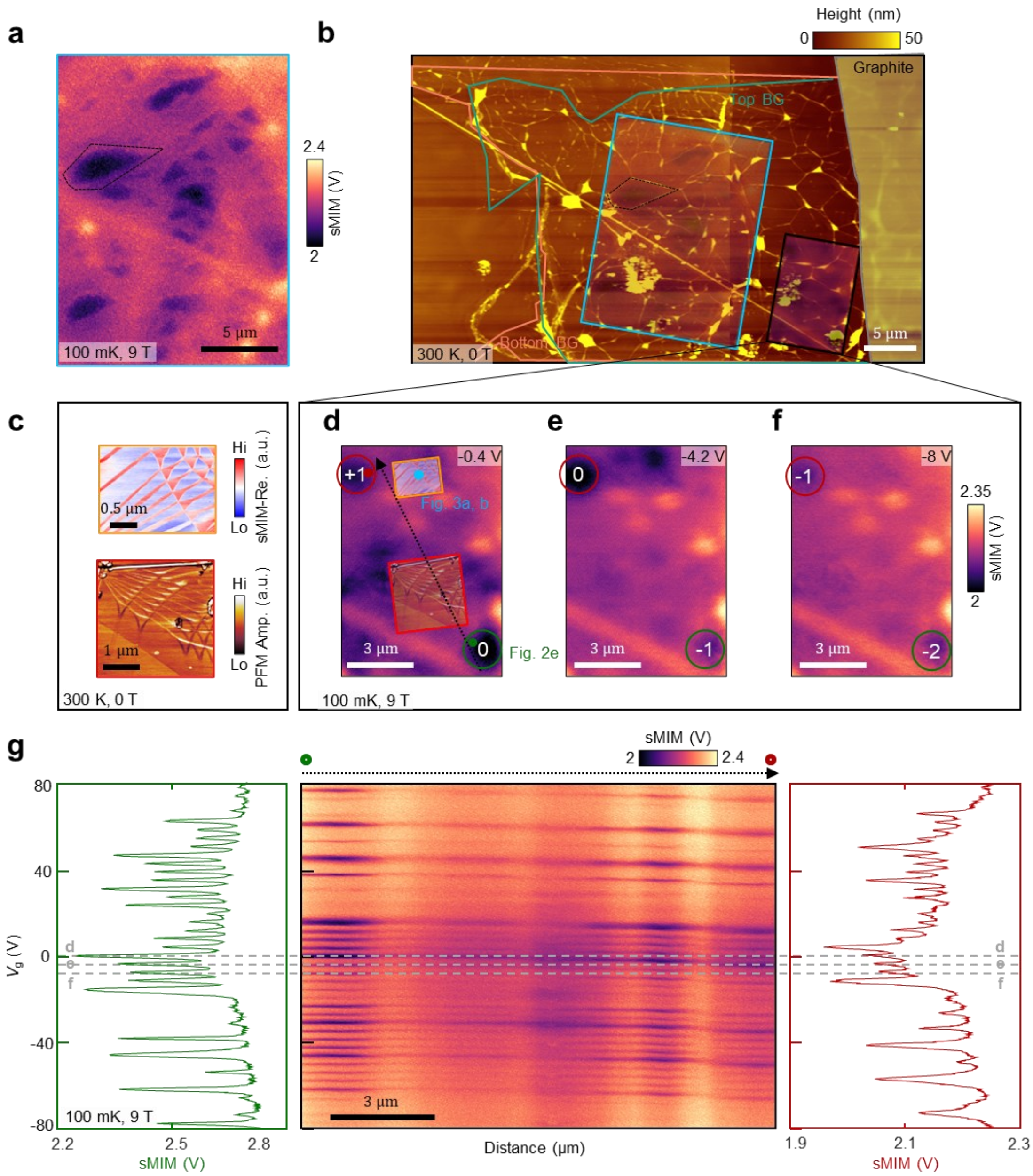

Supplementary Fig. 4 | Coexistence of moiré superlattice and QHIFM states. a, sMIM mapping at V_g = 6.4 V, B = 9 T and T = 100 mK. The incompressible QH states manifest in TDBG interior, confined by structural wrinkles shown in **b**. The dashed black box marks the wrinkle boundary. **b**, AFM topography of device at RT. **c**, Zoom-in sMIM-Re (upper) and PFM amplitude (lower) mapping of moiré superlattice in TDBG at RT. The zoom-in box is indicated in **d**. **d**-**f**, sMIM mapping of TDBG region at V_g = -0.4 V (**d**), -4.2 V(**e**), -8 V(**f**), B = 9 T and T = 100 mK. Domains with different Chern numbers (local Chern numbers are denoted by the numbers) are formed due to local variation of chemical potential. The sMIM measurements (Fig. 2**e** and Fig. 3**a**, **b)** are performed at blue and green dot in **d**. We therefore confirm the coexistence of moiré superlattice and QHIFM states. **g**, sMIM point spectroscopy (left and right) and linecut (middle) at B = 9 T and T = 100 mK along the dashed arrow in **d**. The linecut goes across the moiré superlattice region and maintains a robust QHIFM ground state. The Chern number of green and red dots are shifted by 1 due to local chemical potential difference.

Supplementary Figure 5

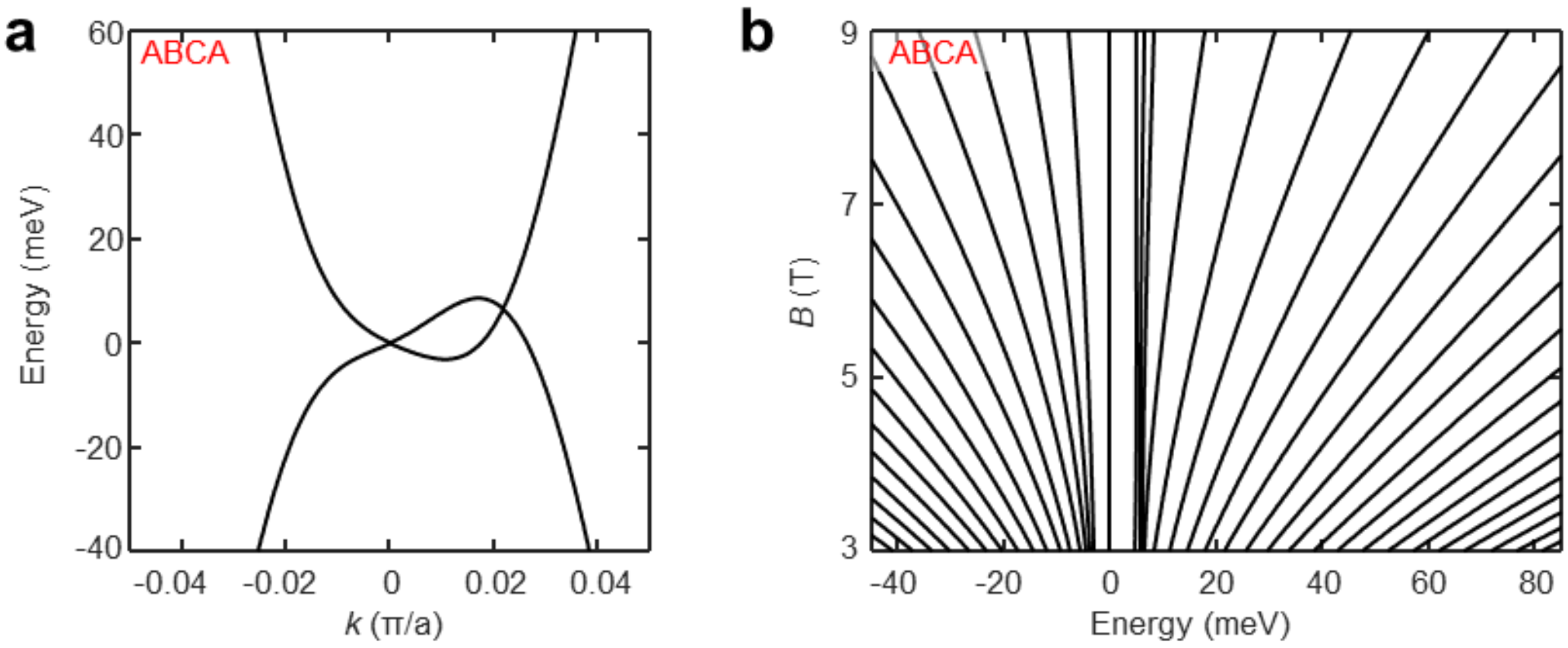

Supplementary Fig. 5 | Theoretical Results of ABCA-stacked tetralayer graphene. a, The calculated energy-momentum dispersion of ABCA graphene. **b**, LL energy spectrum of ABCA graphene. The energy spectrum exhibits absence of LL crossings, which mismatches with experimental results.

Supplementary Figure 6

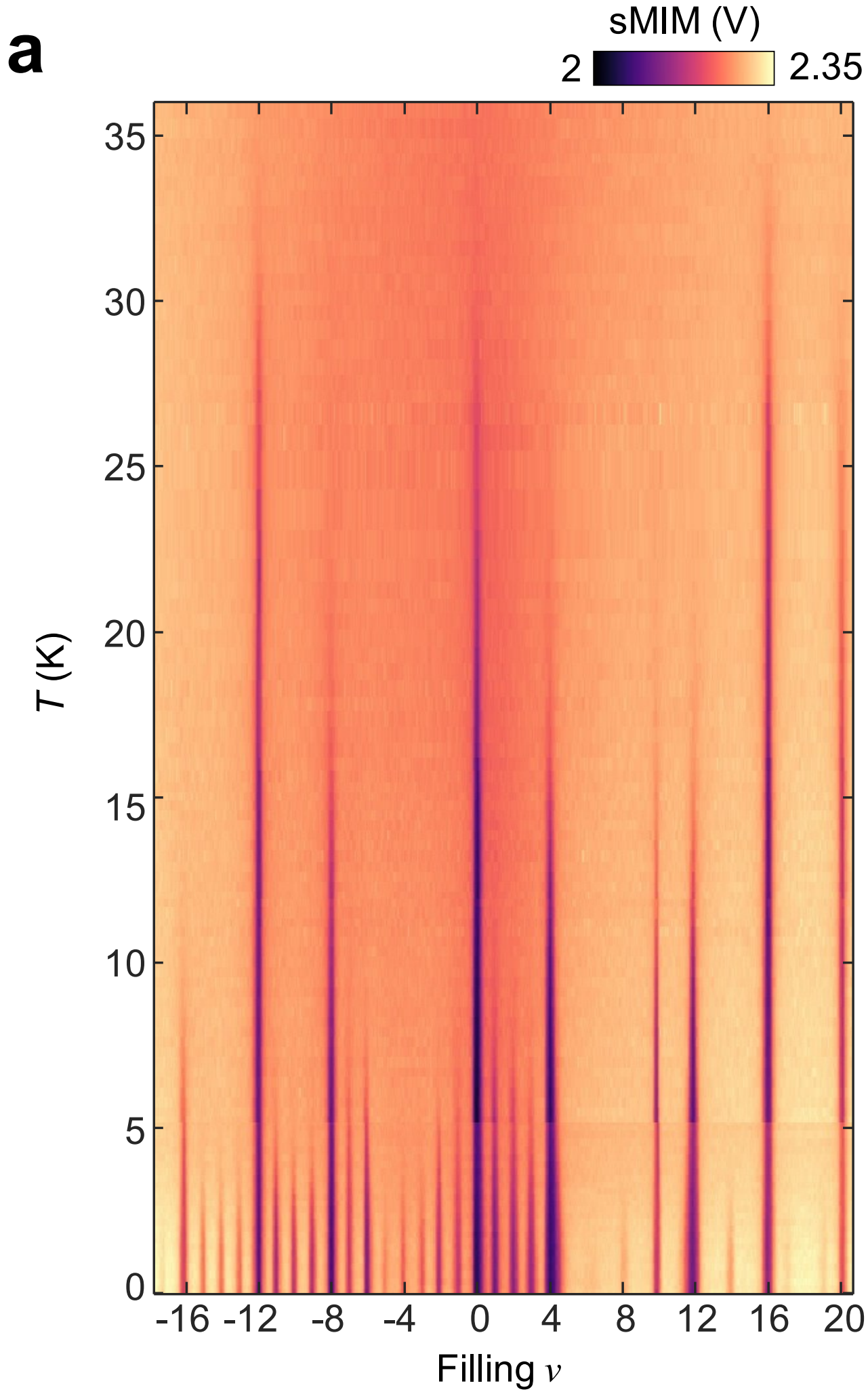

Supplementary Fig. 6 | Temperature dependent sMIM measurement. a, Color plot of temperature dependent sMIM measurement at B = 9 T, shows the robustness of QHIFM states. As the temperature increases, the QHIFM states gradually smear and ultimately vanish at critical temperatures T_C. These critical temperatures are summarized in Fig. 3**e**.

Supplementary Figure 7

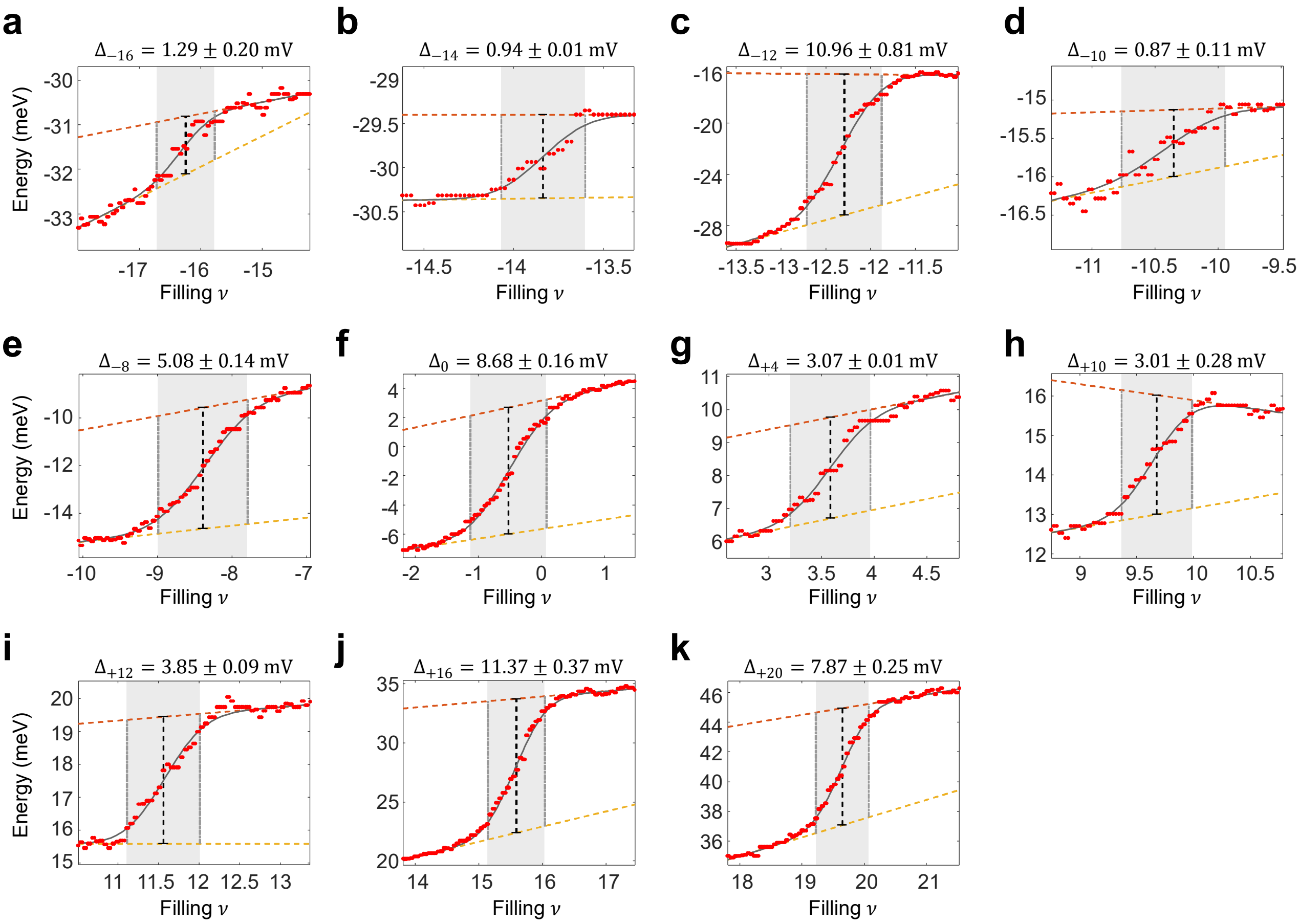

Supplementary Fig. 7 | Extraction of thermodynamic gap. a-k, Chemical potential variation across filling factor -16, -14, -12, -10, -8, 0, +4, +10, +12, +16 and +20 at B = 9 T and T = 100 mK. The energy gaps and their error bounds are determined through the following procedure. First, the local chemical potential plateaus on both sides of the transition are fitted with linear functions $\mu_L(\nu)$ and $\mu_R(\nu)$ (yellow and orange dashed line) to account for the background density of states[47]. Then, the chemical potential step in the transition region is fitted (dark grey solid line) by $\mu_{fit}(\nu) = \mu_L(\nu) + [\mu_R(\nu) - \mu_L(\nu)] \cdot \left[1/2 + 1/2 \cdot \text{erf}\left((\nu - \nu_c)/w\right)\right]$, where erf is the Gaussian error function, ν_c is the gap center and w is the broadening width. Finally, the energy gap Δ is defined as the vertical separation between the two extrapolated baselines evaluated at ν_c: $\Delta = \mu_R(\nu_c) - \mu_L(\nu_c)$. To quantify the uncertainty arising from the finite width of the transition, we define error bounds based on the full width at half maximum (FWHM, grey shaded area) of the Gaussian broadening. The uncertainty $\delta\Delta$ is calculated as the variation of the gap size within the FWHM interval: $\delta\Delta = \frac{1}{2}|\Delta(\nu_c + \text{FWHM}/2) - \Delta(\nu_c - \text{FWHM}/2)|$.

Supplementary Figure 8

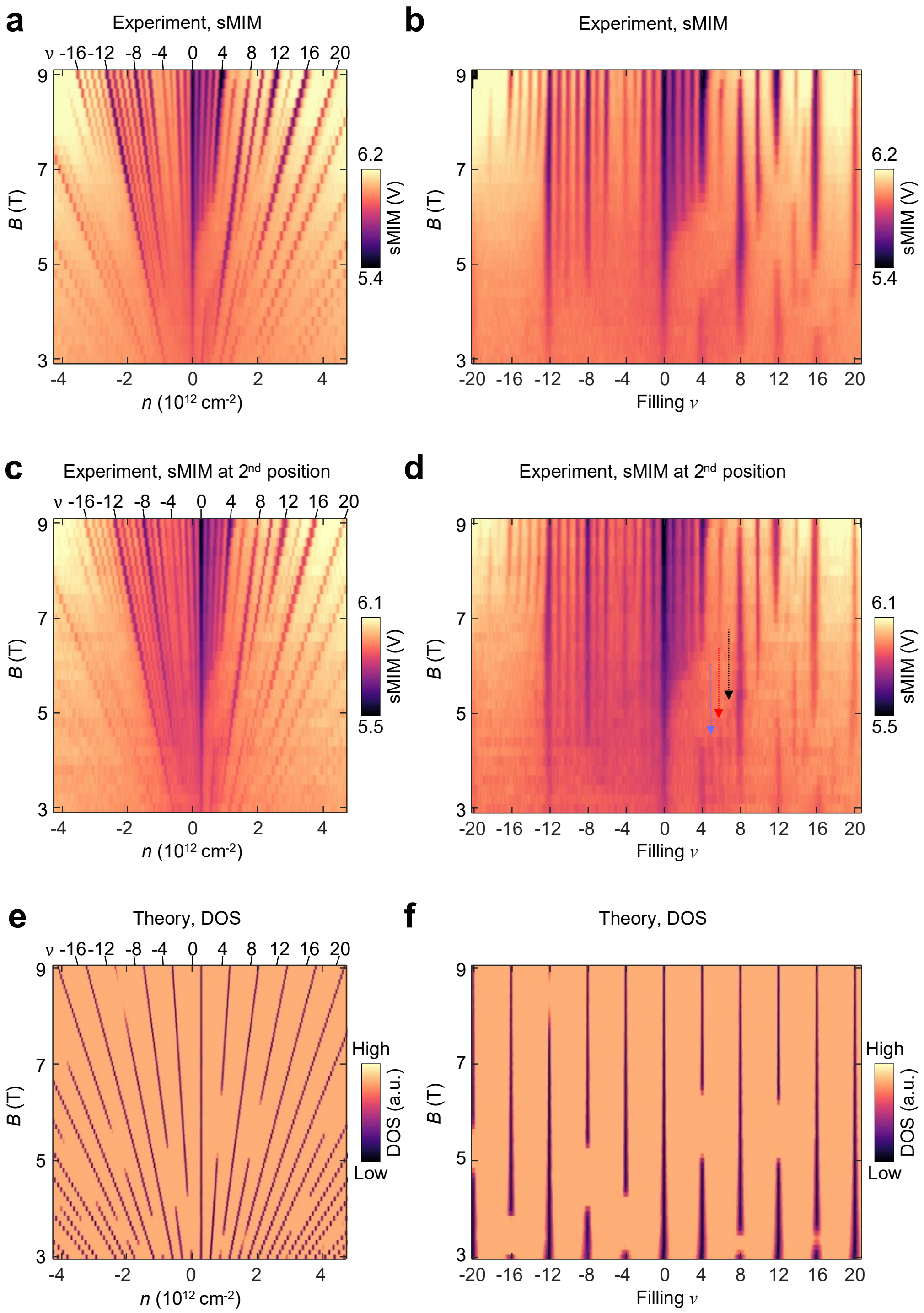

Supplementary Fig. 8 | Comparison between experimental and theoretical results. a, **b**, *n*-*B* map (**a**) and *ν*-*B* map (**b**) of local conductivity at T = 100 mK in TDBG region. **c**, **d**, Same as **a** and **b** while mapping at a different location. The *ν*-*B* map shows an evident symmetry breaking behavior at ν = 5, 6 and 7 below 6 T, as indicated by the arrows in **d**. **e**, **f**, *n*-*B* map (**e**) and *ν*-*B* map (**f**) of theoretically calculated DOS. The LL crossing at ν = 4, 12 and 16 are well reproduced. The calculated results match well with the experimental results.

Supplementary Figure 9

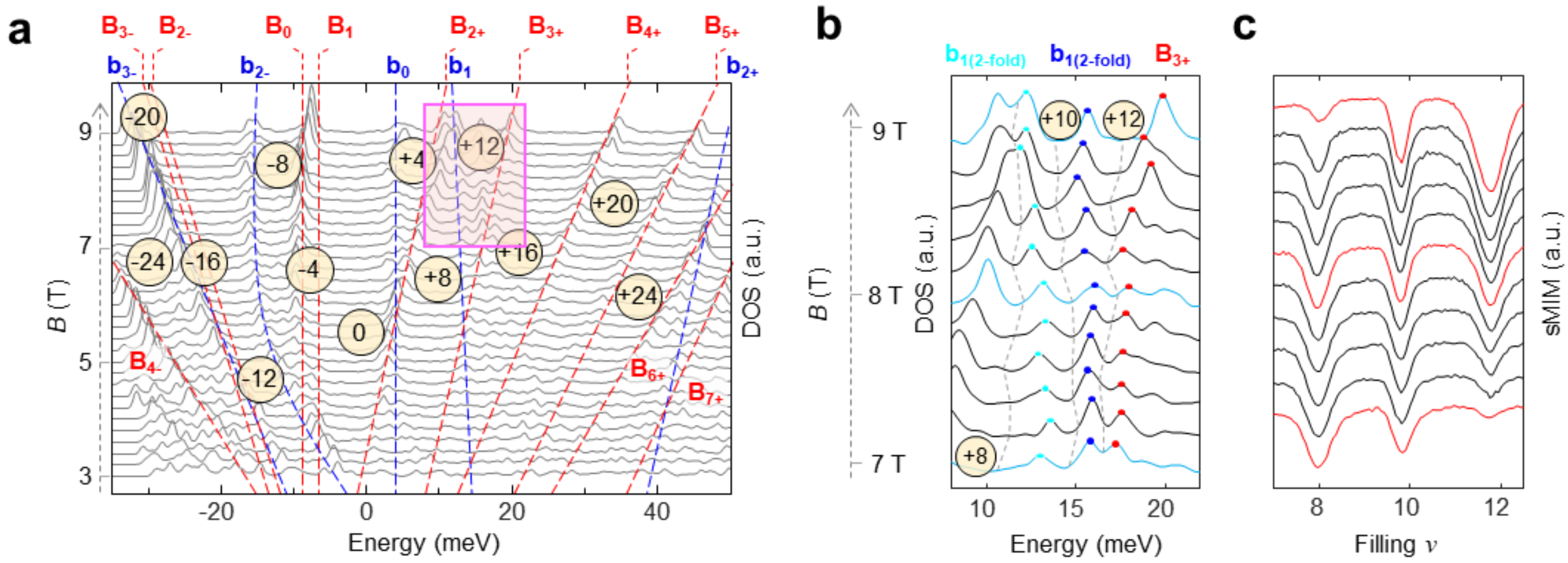

Supplementary Fig. 9. Isospin symmetry breaking within the b_1 LL. **a**, Full LL energy spectrum (same as Fig. 3 c). **b**, Zoom-in LL energy spectrum (magenta box region in **a**) shows the evolution of 2-fold degenerate b_1 LL (cyan and blue circle marked LL) with magnetic field from 7 T to 9 T. Gray dashed lines mark the chemical potential values as a function of magnetic field, corresponding to ν = +8, +10, and +12 QH states. **c**, waterfall plot of sMIM as a function of filling factor ν from 7 T to 9 T. The ν = +10 QH state matches well with the two split sub-peaks in **b**.

Supplementary Figure 10

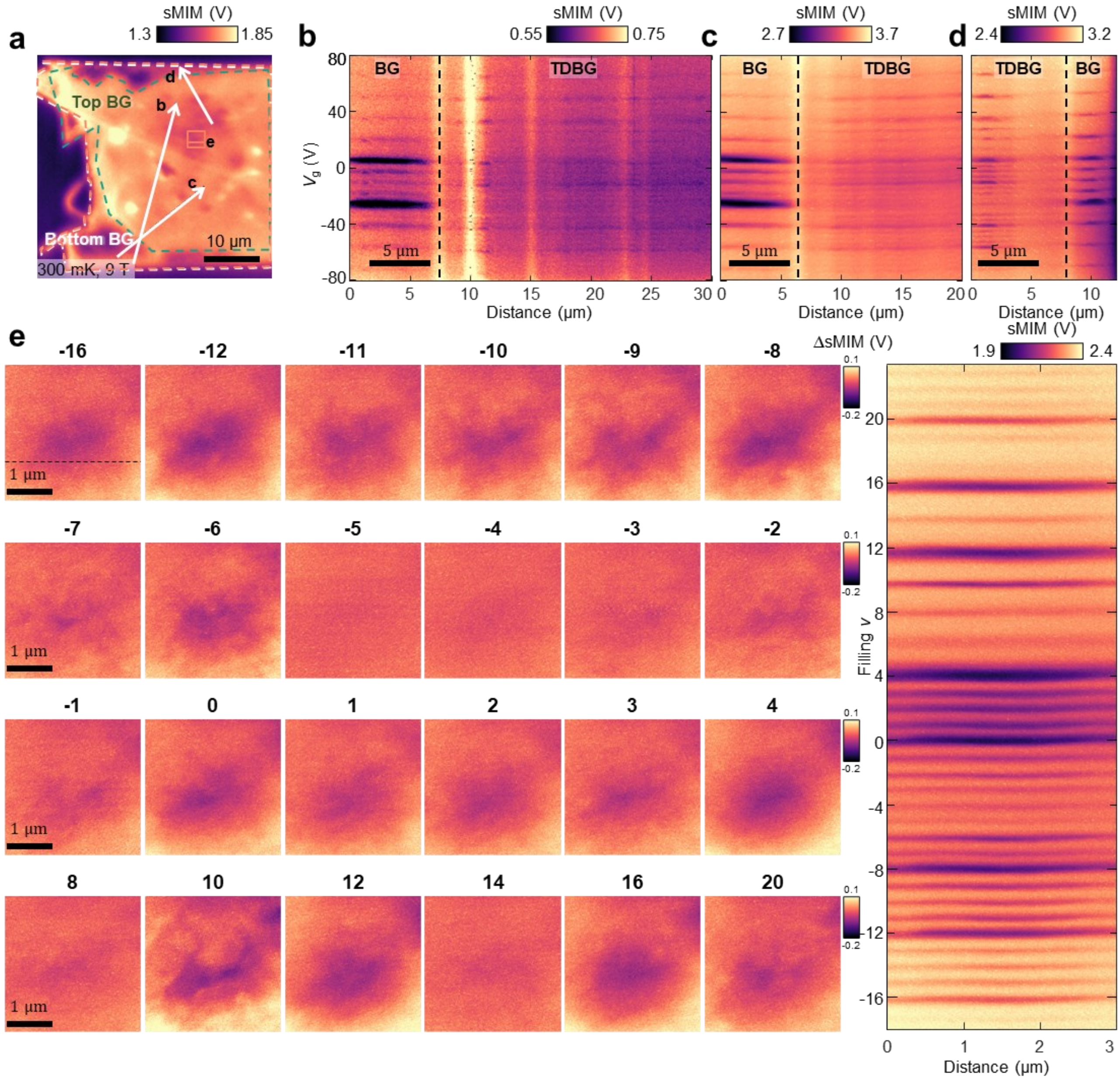

Supplementary Fig. 10. Spatial dependence of the QHIFM states at B = 9 T. a, Large area sMIM mapping of TDBG at B = 9 T and T = 300 mK. The scale bars correspond to 10 μm. The locations of sMIM line scans (**b**, **c**, and **d**) and sMIM-Im mappings (**e**) are indicated by the white arrows and orange box. **b**-**d**, sMIM-Im gate voltage dependent line scans at B = 9 T along the white arrow indicated in **a**. Repeated line scans (y-axis) are performed while the gate voltage is varied (x-axis). **e,** Left: sMIM mappings (B = 9 T and T = 100 mK) of the QH states at different filling factors from -16 to 20. Right: high-resolution sMIM gate voltage dependent line scans along the black dashed line in ν = -16 sMIM mapping.
