# Supplementary material for: Simultaneous nanoscale imaging of local conductivity and chemical potential in a quantum Hall isospin ferromagnet
Source: Nat Commun. 2026 Jun 25;17:7986. doi: 10.1038/s41467-026-74915-y (PMC13448896; doi:10.1038/s41467-026-74915-y)
Supplement: Supplementary file 2 [file 41467_2026_74915_MOESM2_ESM.pdf]

nature portfolio

Peer Review File

Simultaneous nanoscale imaging of local conductivity and chemical potential in a quantum Hall isospin ferromagnet

Corresponding Author: Professor Hong-Jun Gao

This file contains all reviewer reports in order by version, followed by all author rebuttals in order by version.

Version 0:

Reviewer comments:

Reviewer #1

(Remarks to the Author)
The intricate interplay of spin, valley and other degree of freedom in quantum materials necessitates the application of experimental probe capable of sensing different physical properties simultaneously. In this work, the authors introduce one kind of such multi-messenger nanoprobe, so called scanning conductivity and chemical potential microscopy (SCCM), which actually combines scanning microwave impedance microscopy (sMIM) and Kelvin probe force microscopy (KPFM). They demonstrate SCCM by studying quantum Hall (QH) effect in bilayer graphene (BG) and marginally twisted double bilayer graphene (TDBG).

In QH, graphene will develop a series of insulating and incompressible state when its Landau levels (LLs) are fully filled. Graphene in such integer filling condition shows the lowest bulk conductivity and the concurrent chemical potential jump, which can be detected by sMIM and KPFM, respectively. The measured chemical potential shift as a function of filling and magnetic fields in KPFM also allows one to extract the LL energy spectrum and the thermodynamic energy gap of the QH insulating state. These are important physical properties of QH, which can then be directly compared with the sMIM measurement and the theoretically calculated LL spectra.

Technically, the results presented here are appealing. Using BG as a model system, the authors demonstrate the consistency between sMIM and KPFM measurements in characterizing the QH of BG in the sense of LL fan diagram (Fig. 1). The same consistency is observed in Fig. 3e in estimating the energy scale of different QH insulating state. However, notable difference exists between these two methods as shown in Fig. 2e. While the chemical potential jump in KPFM is mostly appreciable at QH states with four-fold degeneracy (v=4N), the conductivity dip in sMIM happens in almost every symmetry-broken QH states (or called quantum Hall isospin ferromagnetic phase, QHIFM). The sensitivity is sort of restored in the thermodynamic density of state (DOS) plot (Fig. 2f) as more peaks appear. As noted by the authors, they “extract the thermodynamic density of states from the chemical potential curve by gaussian kernel density estimation”. Can the authors elaborate on this “gaussian kernel density estimation” method? Given that similar DOS plots shown in Fig. 3 and 4 are the core experimental evidence of the LL crossing, it is essential for readers to know in details how such DOS plot can be extracted from the experimentally measured chemical potential shift in KPFM.

Scientifically, the authors use TDBG as an example to illustrate the power of SCCM to unveil the physical origins of complex quantum phases. Key experimental observations are: 1) symmetry-broken QHIFM states appear in TDBG, but not BG, as shown in Fig. 2d; 2) There is disappearance and re-emergence of certain QHIFM states in TDBG as shown in Fig. 3a; 3) the LL crossing happens in LL energy spectrum as inferred from Fig. 3c. The authors try to argue the connection of LL crossing to the 1st and 2nd observation listed above. For example, the authors say “In contrast, the LL crossing event near v=-8 triggers the emergence of the corresponding QH state above B=4.4 T…..The LL crossing at intermediate magnetic field manifests as an enhancement in local conductivity and a simultaneous closure of the energy gap, demonstrating the remarkable consistency between these two observables” I have several comments regarding the major claims of this part that should be appropriately addressed.

1)Regarding the observation of LL crossing in Fig. 3c, there are still obvious discrepancies between the calculated LL energy spectrum and the DOS plot extracted from the KPFM measurement. For examples, there are several DOS peaks below “+12” round denotation that don’t align with the neighboring b1 or B3+ line. Also, b2- is a curved line in Fig. 3c to be aligned with the DOS peaks. However, b2- should have been a straight line as shown in Fig. 3d which is dictated by

equation (1). The curved feature of DOS peaks becomes more apparent in Fig. 4b and c, which cannot reconcile with the calculated LL spectra in Fig. 3d. I also notice that, the linear dependence of DOS peak energy position on the magnetic field is properly retained in BG as shown in Fig. 1h. What could be the source of such discrepancy in TDBG device?

2)The authors attribute the LL crossing of TDBG to domains with ABAB stacking, which is further confirmed by their band structure calculation. On the other hand, the authors calculate the LL spectra of ABCA-stacked tetralayer graphene which shows no LL crossing. The discrepancy I mentioned above actually lowers the credibility of their claim of LL crossing. To further strengthen it, the authors should perform SCCM on domains with ABCA stacking, and compare the results to the existing ones. I notice that, the authors say “although ABCA-stacked domains are also present, their fraction appears too small to contribute appreciably to the measured signals, rendering their associated features undetectable in our experiment” However, the SCCM measurement is already a spatially resolved point spectrum. If the authors were able to identify both ABAB- and ABCA- stacked domain in conventional AFM imaging (Extended Data Fig. 3), they should try such SCCM point spectrum within the ABCA-stacked domain. If they cannot, an alternative approach is to perform SCCM in naturally existing tetralayer graphene with either ABAB- or ABCA- stacking. It seems to me that, the LL crossing is just a property of multilayer graphene as discussed in ref. 41 (PRB, 83, 165443 (2011)), and doesn’t reply on the formation of Moire domains created in TDBG. I also found no consideration of Moire domains in their theoretical calculation. Therefore, it is informative to compare SCCM results on ABCA-stacked and ABAB-stacked tetralayer graphene. By performing such experiments, one can directly assess the capability of SCCM to resolve LL crossing in energy spectrum. One may also gain insights to the discrepancy in current TDBG device, which could be related to the twisting-induced disorders.

3)An important observation is the formation of symmetry-broken QHIFM states in TDBG, which are absent in neighboring BG area. Such QHIFM states also shows strong electron-hole asymmetry. The emergence of QHIFM states has to do with various spin or valley symmetry breaking process, which is apparently connected to the formation of Moire domains in TDBG device. The current experiment doesn’t reveal much information about the microscopic origins of such symmetry breaking process, such as its relation to the Moire domain structure. On the other hand, the authors try to argue the connection of such QHIFM states to the LL crossing, which is not very convincing for me. As shown in Extended Data Fig. 8d, such QHIFM states (v=5,6,7) exist before and after the LL crossing. The event of LL crossing only shifts the position of QHIFM states, but is not its origin.

4)The role of TDBG for their experimental observations is vaguely discussed. Apparently, such TDBG creates complex structural domain networks and other disordering factors (see Extended Data 3 and 4). Will such Moire superlattice be crucial for the LL crossing and resulting re-emergence of QH states? I doubt. The experiments I suggest in my 2nd comment can answer the question. Will this Moire superlattice in TDBG be crucial for the emergence of QHIFM states? It could be, but the discussion is very limited. The authors should properly discuss the role of TDBG (Moire superlattice) in their observation, so readers can understand the universality of the reported phenomena.

Please address all the concerns raised before this manuscript can be considered for the publication.

Reviewer #2

(Remarks to the Author)
In this paper, J. Hu et al. measured many-body Landau Level states in the moire system of multilayer graphene using an SCCM technique that combines KPFM and sMIM. By implementing feedback at three independent frequencies with three lock-in amplifier setups, the authors simultaneously measured and controlled AFM, KPFM (chemical potential), and sMIM (local conductivity) signals. Through this approach, they extracted the energy gap and DOS of Landau levels in TDBG and BG systems, ultimately completing the phase diagram of QHIFM. Beyond the methodological improvements, this study extends previous scanning probe microscopy investigations - previously limited to conventional 2D Landau systems in mono/bilayer graphene - to moire quantum Hall systems. It is a noteworthy point that the change of DOS with respect to the magnetic field was shown to be consistent with the calculation from Koshino, et al. (2011), and the occurrence of Landau level crossing in a specific range was clearly explained.

Overall, this paper is well-written, and presents a generally smooth logical flow, with clear terminology. However, I believe the following points require revision or further clarification.
In Fig 2b-c, the temperature indicated in the panel (100mK) is not consistent with the caption (300mK).
Correction required: [Line 202] ‘Fig. 3a’ ‘Fig. 3b’
Some of the 2D data plots in the paper lack colorbar labels and units. Without color labels, it's difficult to intuitively understand what the plot represents at a glance, and without unit indicators, quantitative interpretation of the data becomes challenging. Even if arbitrary units are used, they should be stated clearly. (e.g. Fig. 1f-g, Fig. 3a-b)
The explanation of the ‘B, b’ band notation used in Koshino et al. (2011) is not detailed enough in the main text, leading to a lack of clarity. Referring to and explaining Fig. 4(d) inset earlier in the text would help clarify for readers.
In Extended Data Figure 3 (a), the shape of the marked area in the optical photo does not match the shape of the AFM topography boundary. And the sizes of the marked area in the AFM topography do not match the sizes of (c)-(f).
In Extended Data Figure 7, it is not clear how the energy gap was extracted from the chemical potential curve. The energy gap appears to be taken from the difference between the extrapolated lines of the two ends at the center value, but this center value does not always correspond exactly to the desired (even) integer filling. The choice of the fitting range also appears to be inconsistent (absolute/relative). Stating error/adding error bar may help.

And this is my personal opinions regarding color selection.

In most of the 2-dimensional data plots, binary colormaps such as 'bwr' were used. Unless the data has a clear polarity or represents an on/off state where binary colors are particularly meaningful, this type of binary colormap can hinder intuitive interpretation. Selecting a colormap that the contrast changes monotonically, or more intuitively perceptual colormap would improve readability. (e.g., Fig. 3b, Ext Fig. 8)
In Fig. 4c, the symbols used for the middle energy gap and local conductivity have the same color and shape as the peak markers on the left, although they do not share the same physical meaning. I suggest changing the color of these symbols.

These are personal questions and suggestions for future research that were not covered in this paper.
Have you attempted to verify whether the incompressible band generated by the isospin FM state is stronger and more uniform in the bulk rather than at the edge, and if it is generated uniformly at other edges as well?
When extending from the BG to the hBN region, do the local variations of the isospin states near the edge show consistency with the results of previous studies?
Will it be possible to more clearly confirm the continuity of the isospin FM state between BG and TDBG?

The methodological approach of this study is novel and has yielded results that could significantly influence future QHIFM research. With revisions and improvements to the points mentioned above, this paper will be suitable for publication in Nature Communications.

Reviewer #3

(Remarks to the Author)
The manuscript by Hu et al, presents a novel scanning technique that integrates scanning microwave impedance microscopy (sMIM) and Kelvin probe force microscopy (KFM) for probing correlated topological states in low-dimensional materials. The authors first establish the working principle of their techniques in Bernal bilayer graphene and observe the canonical generalized quantum hall ferromagnetic states and extract its Landau level spectrum. Next, they applied their technique to explore the quantum hall states in marginally twisted double bilayer graphene. The Landau fan obtained from the sMIM exhibits several missing sequences due to Landau level crossings. The authors were able to extract the detailed origin of these crossings thanks to the reconstructed many-body Landau level spectrum from the KFM mode. They further analyze these features and speculate on the properties of the Landau level wavefunctions that are most consistent with their data.

Overall, the data quality is very high, and the paper is well written. The technique introduced here will be a useful addition to the scanning probe community for probing the growing class of interesting van der Waals heterostructures. I recommend publication in Nature Communications provided that the authors address my following comments.
1. Are the sMIM and KFM operations truly simultaneous?
2. Why there are more incompressible states near CNP in the TDBG region than in the BG region?
3. I suggest labeling the peaks in Fig. 2f with their Landau level index.

Version 1:

Reviewer comments:

Reviewer #1

(Remarks to the Author)
In this revised manuscript, the authors have adequately addressed all concerns raised in my previous review. In particular, they provided a detailed description of the “gaussian kernel density estimation” method. They also explained the physical origin of the discrepancy between the experimentally observed curved b2- line and the theoretically calculated straight one. Moreover, they clarified the “Moireless” nature of their TDBG device. I believe the SCCM method (sMIM&KPFM) introduced in this work will benefit the research in topological phase of matters especially found in vdW Moire systems, and recommend to publish this paper in Nature Communications.

Reviewer #2

(Remarks to the Author)
This revision has adequately addressed all of my comments and requested corrections. In the revised manuscript, the content is clear and flows smoothly, and I could not identify any further points for improvement. I appreciate the authors' response to all of my comments and incorporation of the requested changes into the manuscript.

Reviewer #3

(Remarks to the Author)
The authors have adequately addressed the comments raised by me and other reviewers. I recommend publication in Nature Communications.

Open Access This Peer Review File is licensed under a Creative Commons Attribution 4.0 International License, which permits use, sharing, adaptation, distribution and reproduction in any medium or format, as long as you give appropriate credit to the original author(s) and the source, provide a link to the Creative Commons license, and indicate if changes were made.
In cases where reviewers are anonymous, credit should be given to 'Anonymous Referee' and the source.
The images or other third party material in this Peer Review File are included in the article's Creative Commons license, unless indicated otherwise in a credit line to the material. If material is not included in the article's Creative Commons license and your intended use is not permitted by statutory regulation or exceeds the permitted use, you will need to obtain permission directly from the copyright holder.
To view a copy of this license, visit https://creativecommons.org/licenses/by/4.0/

Summary of main changes

We sincerely appreciate the positive evaluation of our paper and insightful comments provided by all the reviewers, which have significantly enhanced the quality and clarity of our manuscript. In response to the constructive feedback, we have provided a point-by-point response to each reviewer's remarks **in blue** and made revisions in the manuscript and figures highlighted **in red**. Following the constructive suggestions of the reviewers, the main changes to the manuscript and figures are summarized as follows:

1. Following Reviewer #1's suggestion, we have added a comprehensive description of the gaussian kernel density estimation method in the Methods section

2. In response to Reviewer #1's comments, we have added several new figures: Extended Data Fig. 3g–n (additional moiré regions), Extended Data Fig. 9 (multi-peaks feature at ν = +12), and Extended Data Fig. 10 (ubiquitous QHIFM phases and moiré-induced textures). We also revised the main text to explain the coexistence of moiré and moiré-free regions and clarified the mechanical limitations that prevented in situ imaging of distinct stacking domains.

3. In response to Reviewer #1 and #3's comments, we have added new discussions on the curved feature of b_{2-}, electron-hole asymmetry, and the difference of QHIFM states between BG and TDBG. These are included in the main text as requested.

4. Following Reviewer #1's suggestions, we have added a discussion section that supports the dominant ABAB phase using recent transport work and clarifies the effect of LL crossing on the QHIFM phase diagram to avoid ambiguity.

5. Following Reviewer #2's suggestions, we have added a detailed description of the origin of the light and heavy BG bands and revised the single-particle LL wavefunction part to clarify the multi-component nature of LL subbands.

6. In response to Reviewer #2's and Reviewer #3's comments, we have revised several figures: Fig. 2f (explicit Landau level indices), Extended Data Fig. 3 (AFM and sMIM-Re mappings), and Extended Data Fig. 7 (gap extraction procedure and error bars). All updates are reflected in the manuscript and captions.

7. Following Reviewer #2's suggestions, explicit colorbar labels with actual physical units (e.g., V, meV) have been added to all sMIM and KPFM data plots. All diverging binary colormaps in the 2D data plots have been completely replaced with perceptually uniform, monotonic colormaps ('magma' and 'viridis') throughout the revised manuscript and extended data

Point-by-point response to reviewers

Response to reviewer #1

The intricate interplay of spin, valley and other degree of freedom in quantum materials necessitates the application of experimental probe capable of sensing different physical properties simultaneously. In this work, the authors introduce one kind of such multi-messenger nanoprobe, so called scanning conductivity and chemical potential microscopy (SCCM), which actually combines canning microwave impedance microscopy (sMIM) and Kelvin probe force microscopy (KPFM). They demonstrate SCCM by studying quantum Hall (QH) effect in bilayer graphene (BG) and marginally twisted double bilayer graphene (TDBG).

In QH, graphene will develop a series of insulating and incompressible state when its Landau levels (LLs) are fully filled. Graphene in such integer filling condition shows the lowest bulk conductivity and the concurrent chemical potential jump, which can be detected by sMIM and KPFM, respectively. The measured chemical potential shift as a function of filling and magnetic fields in KPFM also allows one to extract the LL energy spectrum and the thermodynamic energy gap of the QH insulating state. These are important physical properties of QH, which can then be directly compared with the sMIM measurement and the theoretically calculated LL spectra.

# Reply:

We sincerely appreciate the reviewer's thorough evaluation and constructive recommendations. These insightful comments have significantly enhanced the quality and clarity of our manuscript.

# Question 1-1:

Technically, the results presented here are appealing. Using BG as a model system, the authors demonstrate the consistency between sMIM and KPFM measurements in characterizing the QH of BG in the sense of LL fan diagram (Fig. 1). The same consistency is observed in Fig. 3e in estimating the energy scale of different QH insulating state. However, notable difference exists between these two methods as shown in Fig. 2e. While the chemical potential jump in KPFM is mostly appreciable at QH states with four-fold degeneracy (v=4N), the conductivity dip in sMIM happens in almost every symmetry-broken QH states (or called quantum Hall isospin ferromagnetic phase, QHIFM). The sensitivity is sort of restored in the thermodynamic density of state (DOS) plot (Fig. 2f) as more peaks appear. As noted by the authors, they “extract the thermodynamic density of states from the chemical potential curve by

gaussian kernel density estimation". Can the authors elaborate on this "gaussian kernel density estimation" method? Given that similar DOS plots shown in Fig. 3 and 4 are the core experimental evidence of the LL crossing, it is essential for readers to know in details how such DOS plot can be extracted from the experimentally measured chemical potential shift in KPFM.

# Reply:

We thank the reviewer for highlighting this important point. We agree that providing a detailed explanation of the DOS extraction method is essential for validating the experimental evidence of LL crossings presented in this work.

In SCCM measurement, the carrier density n varies linearly as the gate voltage changes (uniform sampling). Therefore, the statistical distribution of the measured chemical potential values, $P(\mu)$, is directly proportional to the inverse slope of the $\mu(n)$ curve, which corresponds to the thermodynamic density of states ($\mathrm{DOS}(\mu) = \frac{e^2 dn}{d\mu}$). This means that energy regions where $\mu(n)$ exhibits plateaus (compressible states) accumulate a higher density of data points, resulting in peaks in the DOS.

We specifically chose gaussian kernel density estimation (GKDE) for this extraction rather than direct numerical differentiation, which can amplify high-frequency experimental noise and obscure genuine spectral features. This GKDE method treats each data point as a gaussian wave packet and superimposes them to form the total spectrum. The DOS is proportional to the sum of these gaussian kernels:

$$\mathrm{DOS}(\mu) \propto \frac{1}{Nh}\sum_{i=1}^{N}\frac{1}{\sqrt{2\pi}}exp\left(-\frac{(\mu-\mu_i)^2}{2h^2}\right)$$

where N is the total number of data points and h is the width of the gaussian kernel. This GKDE method serves as a robust statistical filter: it reconstructs the continuous DOS profile by superimposing gaussian kernels, thereby effectively suppressing sampling noise while preserving the integrity of intrinsic features, such as the Landau levels spectrum in Figs. 1 and 3.

# Revision:

1. In the revised manuscript, we have added a comprehensive description of the gaussian kernel density estimation method in the Methods section (Page 21):

   "The thermodynamic density of states, defined as $\mathrm{DOS}(\mu) = \frac{e^2 dn}{d\mu}$, relates to the inverse slope of the measured $\mu(n)$ dispersion. In our experiment, the linear sweep of gate voltage ensures uniform sampling intervals in carrier density n. Consequently, the statistical distribution of the measured μ values directly reflects

the DOS structure: energy regions where $\mu(n)$ is flat accumulate a higher density of data points, corresponding to peaks in the DOS.

To reconstruct a continuous DOS spectrum from the discrete experimental data points $\{\mu_i\}$, we employed the gaussian kernel density estimation method. This approach effectively treats each data point as a gaussian wave packet and superimposes them to form the total spectrum. The estimated DOS is proportional to the sum of these gaussian kernels:

$$\mathrm{DOS}(\mu) \propto \frac{1}{Nh}\sum_{i=1}^{N}\frac{1}{\sqrt{2\pi}}exp\left(-\frac{(\mu-\mu_i)^2}{2h^2}\right)$$

where N is the total number of data points and h is the width of the gaussian kernel. The width h was carefully selected based on the experimental data quality to achieve an optimal balance between suppressing measurement noise and resolving fine spectral features (such as Landau level splitting). Specifically, we use a width of h = 0.5 meV for the BG data and h = 0.35 meV for the TDBG data. Compared to direct numerical differentiation which amplifies high-frequency experimental noise, this statistical approach yields a smooth, continuous DOS profile that allows for the unambiguous identification of intrinsic energy gaps and Landau levels."

2. We have revised the sentence in Page 6, line 120: "We extract the thermodynamic density of states (DOS) from the chemical potential curve by gaussian kernel density estimation (Fig. 1e, see "Methods"), wherein the LLs manifest as a series of peaks situated at discrete energies."

# Question 1-2:

Scientifically, the authors use TDBG as an example to illustrate the power of SCCM to unveil the physical origins of complex quantum phases. Key experimental observations are: 1) symmetry-broken QHIFM states appear in TDBG, but not BG, as shown in Fig. 2d; 2) There is disappearance and re-emergence of certain QHIFM states in TDBG as shown in Fig. 3a; 3) the LL crossing happens in LL energy spectrum as inferred from Fig. 3c. The authors try to argue the connection of LL crossing to the 1st and 2nd observation listed above. For example, the authors say "In contrast, the LL crossing event near v=-8 triggers the emergence of the corresponding QH state above B=4.4 T…..The LL crossing at intermediate magnetic field manifests as an enhancement in local conductivity and a simultaneous closure of the energy gap, demonstrating the remarkable consistency between these two observables" I have several comments regarding the major claims of this part that should be appropriately addressed.

1)Regarding the observation of LL crossing in Fig. 3c, there are still obvious discrepancies between the calculated LL energy spectrum and the DOS plot extracted from the KPFM measurement. For examples, there are several DOS peaks below "+12" round denotation that don't align with the neighboring b1 or B3+ line.

# Reply:

We thank the reviewer for the keen observation of the detailed spectral features in Fig. 3c. We have performed a detailed analysis of the specific region mentioned and provide the following clarification on the DOS peaks below the $\nu = +12$ denotation.

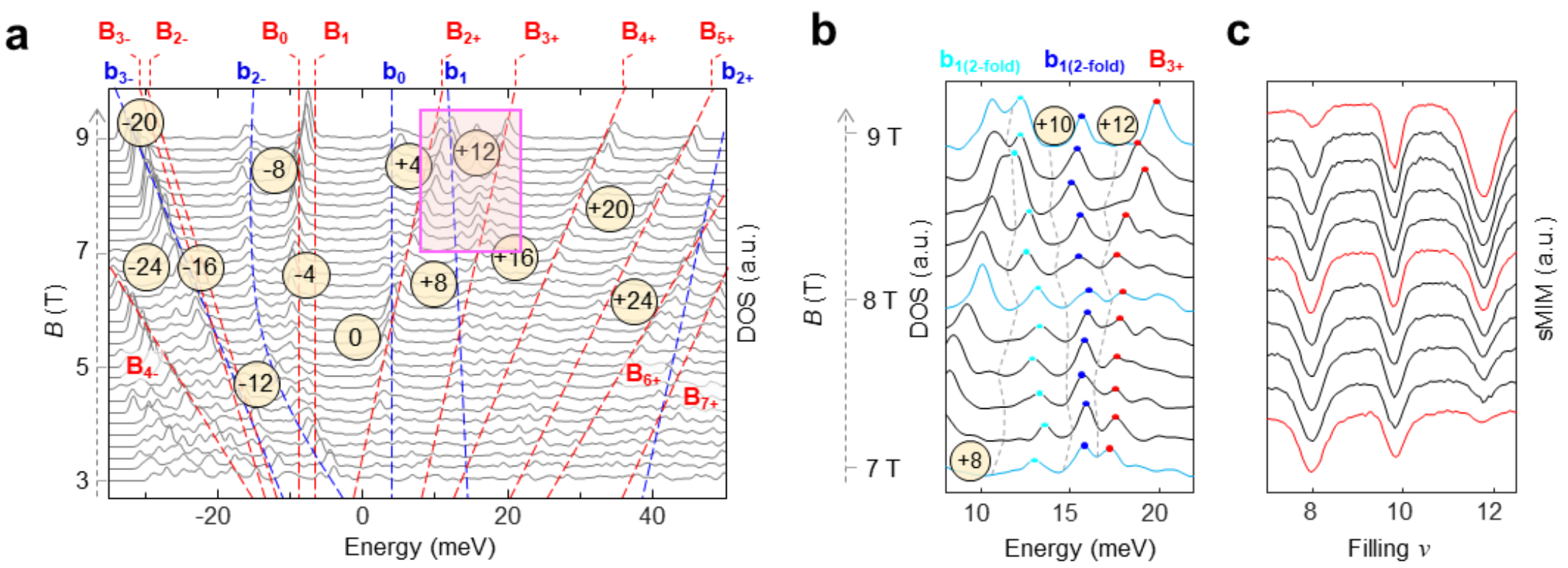

Figure R1. Isospin symmetry breaking within the b_1 LL. a, Full LL energy spectrum (same as Fig. 3 c). **b**, Zoom-in LL energy spectrum (magenta box region in **a**) shows the evolution of 2-fold degenerate b_1 LL (cyan and blue circle marked LL) with magnetic field from 7 T to 9 T. Gray dashed lines mark the chemical potential values as a function of magnetic field, corresponding to $\nu = +8$, +10, and +12 QH states. **c**, waterfall plot of sMIM as a function of filling factor ν from 7 T to 9 T. The $\nu = +10$ QH state matches well with the two split sub-peaks in **b**.

The reviewer noted that the experimental DOS peaks near the $\nu = +12$ denotation do not align with a simple b_1 and B_{3+} line. This is due to the resolved isospin symmetry breaking behavior of the b_1 LL both in local conductivity and chemical potential channel. As shown in the Fig. R1b (zoom-in of the magenta box region), the 4-fold degenerated b_1 LL splits into two distinct 2-fold degenerated sub-peaks (marked by cyan and blue circles). To verify this, we carefully track the chemical potential as a function of magnetic field at $\nu = +8$, +10 and +12 QH states (gray dashed lines in Fig. R1b). It is evident that the $\nu = +10$ QH energy gap aligns perfectly with the dip between these two split sub-peaks. In the meanwhile, we observe a clear $\nu = +10$ QH state in local conductivity channel (Fig. R1c). Therefore, the complex peak structure observed experimentally is not a misalignment but a result of isospin symmetry breaking within the b_1 LL, a feature beyond the reach of non-interacting models that our SCCM technique has sufficient resolution to resolve.

The origin of this $\nu = +10$ QH state could be the valley-splitting of the b_1 band induced by the perpendicular electric field. Specifically, based on our calculations, the K-valley wavefunction of b_1 is localized at the at β_2 and β_4 sites, while the K'-valley wavefunction is localized at $\alpha_1$and α_3 sites. Under a positive gate voltage (which breaks the inversion symmetry), the interlayer potential difference introduces an energy splitting between these layer-polarized valley states, giving rise to the two 2-fold spin degenerated sub-bands observed in the experiment (Fig. R1b). However, exact identification of the nature of this state requires layer-sensitive experimental techniques like penetration field capacitance measurement (Ref. 4) or STM (Refs. 8, 9 and 33).

# Revision:

1. To clarify the origin of the complex DOS peaks in Fig. 3 c around $\nu = +12$, we have added Fig. R1 as Extended Data Fig. 9 in the revised manuscript.
2. A new sentence has been added to clarify the multi-peaks feature around $\nu = +12$ (page 16, line 320): "Such valley symmetry splitting is clearly resolved by the multi-peaks feature in the zoomed-in LL energy spectrum near ν = +12 (Extended data Fig. 9), where the 4-fold degenerate b_1 LL splits into two distinct 2-fold degenerate sub-peaks. This splitting is consistent with a clear $\nu = +10$ QH state in corresponding local conductivity channel."

# Question 1-3:

Also, b2- is a curved line in Fig. 3c to be aligned with the DOS peaks. However, b2- should have been a straight line as shown in Fig. 3d which is dictated by equation (1). The curved feature of DOS peaks becomes more apparent in Fig. 4b and c, which cannot reconcile with the calculated LL spectra in Fig. 3d. I also notice that, the linear dependence of DOS peak energy position on the magnetic field is properly retained in BG as shown in Fig. 1h. What could be the source of such discrepancy in TDBG device?

# Reply:

The reviewer correctly points out that the experimental dispersion of b_{2-} is curved, deviating from the linear prediction of Eq. (1), whereas the BG data retains a linear dependence. We attribute this discrepancy to the fundamental difference between the thermodynamic chemical potential measured in our experiment and the single-particle energy band calculated in theory.

The theoretical lines in Fig. 3d represent the LL energy spectrum of non-interacting electrons. The single-particle LL energy is dominated by the cyclotron energy $\hbar\frac{eB}{m^*}$, which scales linearly with the magnetic field (as dictated by Eq. 1). In contrast, our

experiment measures the chemical potential $\mu(n)$ and thermodynamic DOS. In a strongly correlated system like QHIFM states, adding an electron involves overcoming not only the single-particle energy gap, but also the many-body exchange energy associated with the polarized QHIFM states. The exchange energy gap is governed by the Coulomb interaction scale $E_C = \frac{e^2}{\epsilon l_B}$, where the magnetic length $l_B = \sqrt{\frac{\hbar}{eB}}$. Consequently, the interaction contribution scales as $\sqrt{B}$ which will introduce a non-linear contribution to the LLs energy. Therefore, in our actual measurement, the energy position of the b_{2-} peak is not solely determined by the single-particle cyclotron energy gap described in Eq. (1); it is also significantly influenced by the QHIFM states between ν = 0 and ν = -8 driven by electron-electron interactions, leading to the observed deviation of the experimental value from the single-particle calculation.

As shown in Fig. 2d, BG exhibits fewer QHIFM states compared to the dense sequence of broken-symmetry states in TDBG. This implies that the interaction-induced modification to the single-particle bands is relatively weaker in BG than TDBG. We also note that the energy scale of the cyclotron gap in BG is considerably larger, with our chemical potential measurements spanning from -60 meV to 100 meV (Fig. 1h), whereas the TDBG spectrum covers a narrower range of approximately -30 meV to 40 meV (Fig. 3c). In BG, the large linear cyclotron energy term dominates the total energy, rendering the non-linear interaction effects less pronounced.

# Revision:

1. To explain the origin of the curved feature of b_{2-}, we have added a discussion in page 15 line 281: "We note that the experimental b_{2-} band is a curved line in Fig. 4b, which is apparently deviated from a linear dispersion with magnetic field expected by the theoretical result (Fig. 3d). In SCCM measurement, the energy position of the b_{2-} peak is not solely determined by the single-particle cyclotron energy gap described in equation (1); it is also significantly influenced by the QHIFM states between ν = 0 and ν = -8 driven by electron-electron interactions, leading to the observed deviation of the experimental value from the single-particle calculation. Therefore, we attribute this discrepancy to the fundamental difference between the thermodynamic chemical potential measured in our experiment and the single-particle energy band calculated in theory."

# Question 1-4:

2)The authors attribute the LL crossing of TDBG to domains with ABAB stacking, which is further confirmed by their band structure calculation. On the other hand, the authors calculate the LL spectra of ABCA-stacked tetralayer graphene which shows no

LL crossing. The discrepancy I mentioned above actually lowers the credibility of their claim of LL crossing. To further strengthen it, the authors should perform SCCM on domains with ABCA stacking, and compare the results to the existing ones. I notice that, the authors say "although ABCA-stacked domains are also present, their fraction appears too small to contribute appreciably to the measured signals, rendering their associated features undetectable in our experiment" However, the SCCM measurement is already a spatially resolved point spectrum. If the authors were able to identify both ABAB- and ABCA- stacked domain in conventional AFM imaging (Extended Data Fig. 3), they should try such SCCM point spectrum within the ABCA-stacked domain.

# Reply:

We appreciate the reviewer's suggestion to conduct a control experiment on ABCA-stacked domains. While we agree that a direct comparison would be ideal, we encountered technical issues in isolating the ABCA spectrum. Instead, we performed additional sMIM gate dependent large-scale line scans, high-resolution spatial mappings (Fig. R2) and sMIM-Re RT mappings (Extended Data Fig. 3 g-n) to demonstrate that the observed QHIFM states are spatially universal and originate from the dominant ABAB phase.

The reviewer points out that ABCA domains are visible in Extended Data Fig. 3. However, it is important to note that these images were obtained at room temperature using an Asylum Cypher S AFM system, which benefits from excellent environmental isolation and minimal mechanical noise. In contrast, our SCCM measurements were conducted in a dry dilution refrigerator at 100 mK, where large mechanical vibration noise is generated by the pulse tube cryocooler. We have attempted to image the moiré superlattice at 100 mK using both contact (sMIM, conductive-AFM and PFM) and non-contact (sMIM, KPFM and electrostatic force microscopy) modes to locate the ABCA domains. However, despite our best efforts, the mechanical noise level in the dry-DR environment prevented us from resolving the moiré superlattice in situ.

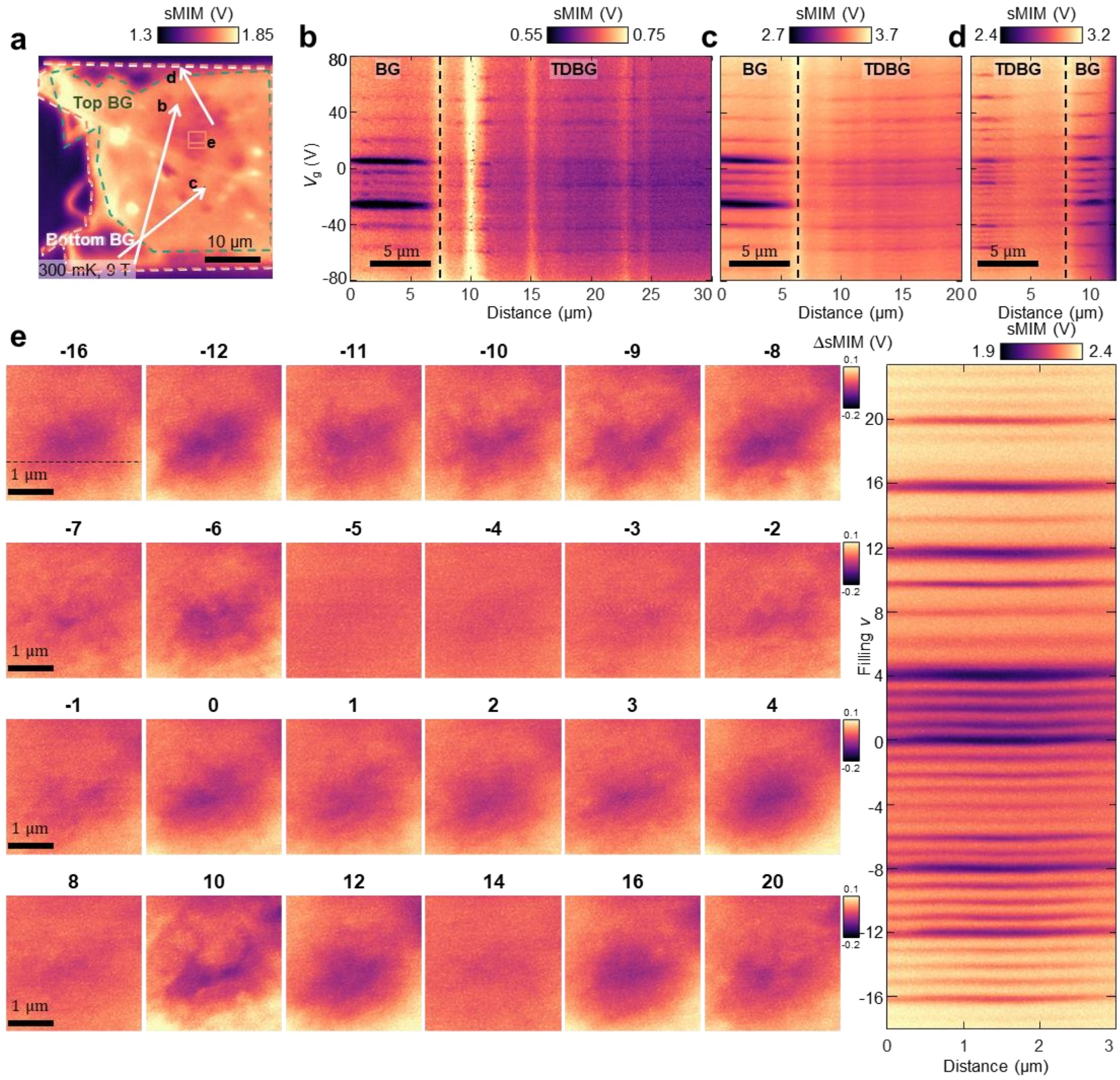

Figure R2. Spatial dependence of the QHIFM states at $B = 9$ T. a, Large area sMIM mapping of TDBG at $B = 9$ T and $T = 300$ mK. The scale bars correspond to 10 μm. The locations of sMIM line scans (**b**, **c**, and **d**) and sMIM-Im mappings (**e**) are indicated by the blue arrows and orange box. **b-d**, sMIM-Im gate voltage dependent line scans at $B = 9$ T along the blue arrow indicated in **a**. Repeated line scans (y-axis) are performed while the gate voltage is varied (x-axis). **e,** Left: sMIM mappings ($B = 9$ T and $T = 100$ mK) of the QH states at different filling factors from -16 to 20. Right: high-resolution sMIM gate voltage dependent line scans along the black dashed line in $\nu = -16$ sMIM mapping.

To rule out ambiguity raised by the reviewer and confirm that our spectroscopic data represents the dominant ABAB phase, we instead performed sMIM line scans across large areas (> 10 μm) spanning from the BG region to the TDBG region (Fig. R2 b-d). These scans reveal the ubiquitous presence of the QHIFM state sequences across the entire TDBG region. We note that the signal amplitude and the exact gate voltage positions of these states vary spatially due to intrinsic charge disorder. However, the characteristic sequence of the QH states remains intact everywhere, consistent with the data in Fig. 2d. We do not observe any regions exhibiting the distinct spectrum expected

for ABCA stacking. We also performed high-resolution sMIM 2D mapping over a 3×3 μm region at various filling factors (Fig. R2e, left). Although the mechanical noise prevented direct imaging of the moiré lattice, we observe subtle spatial patterns near certain QHIFM states (e.g. ν = -7 and +1), which might be the signature of the underlying moiré superlattice. Importantly, the spectroscopic signature of sMIM line scan across this area reveals a clearly resolved and uniform QHIFM sequence (Fig. R2e, right).

To provide a more complete picture of the sample and to further support the dominance of the ABAB phase, we have included additional room-temperature sMIM-Re mappings (panels g–n) in the revised Extended Data Fig. 3, complementing the previously shown moiré regions. These new zoom-in scans reveal that extensive regions within other confined blocks completely lack moiré superlattices, having fully relaxed into a uniform ABAB stacking phase. To highlight the spatial relationship between these domains, we note that the moiré region in panel (f) is directly adjacent to the moiré-free region in panel (j), clearly capturing the structural evolution from a mixed ABCA/ABAB moiré state into a pure ABAB phase. A similar spatial adjacency is also observed between the regions in panel (d) and panel (i). Based on these mappings, we conclude that the ABAB stacking phase robustly dominates the overall device area, regardless of the local presence of a moiré superlattice.

In conclusion, despite local variations, possible moiré-induced textures, and the alternating presence or absence of moiré superlattices, the consistent observation of the characteristic QHIFM sequence across the entire sample confirms that the signal is robustly dominated by the ABAB phase.

# Revision:

1. To better explain the ubiquitous QHIFM phases across the sample and possible moiré-induced textures, we have added Fig. R2 as Extended Data Fig. 10 in the revised manuscript.
2. In the revised Extended Data Fig. 3, we have included additional room-temperature sMIM-Re mappings (panels g–n) to complement the previously shown moiré regions.

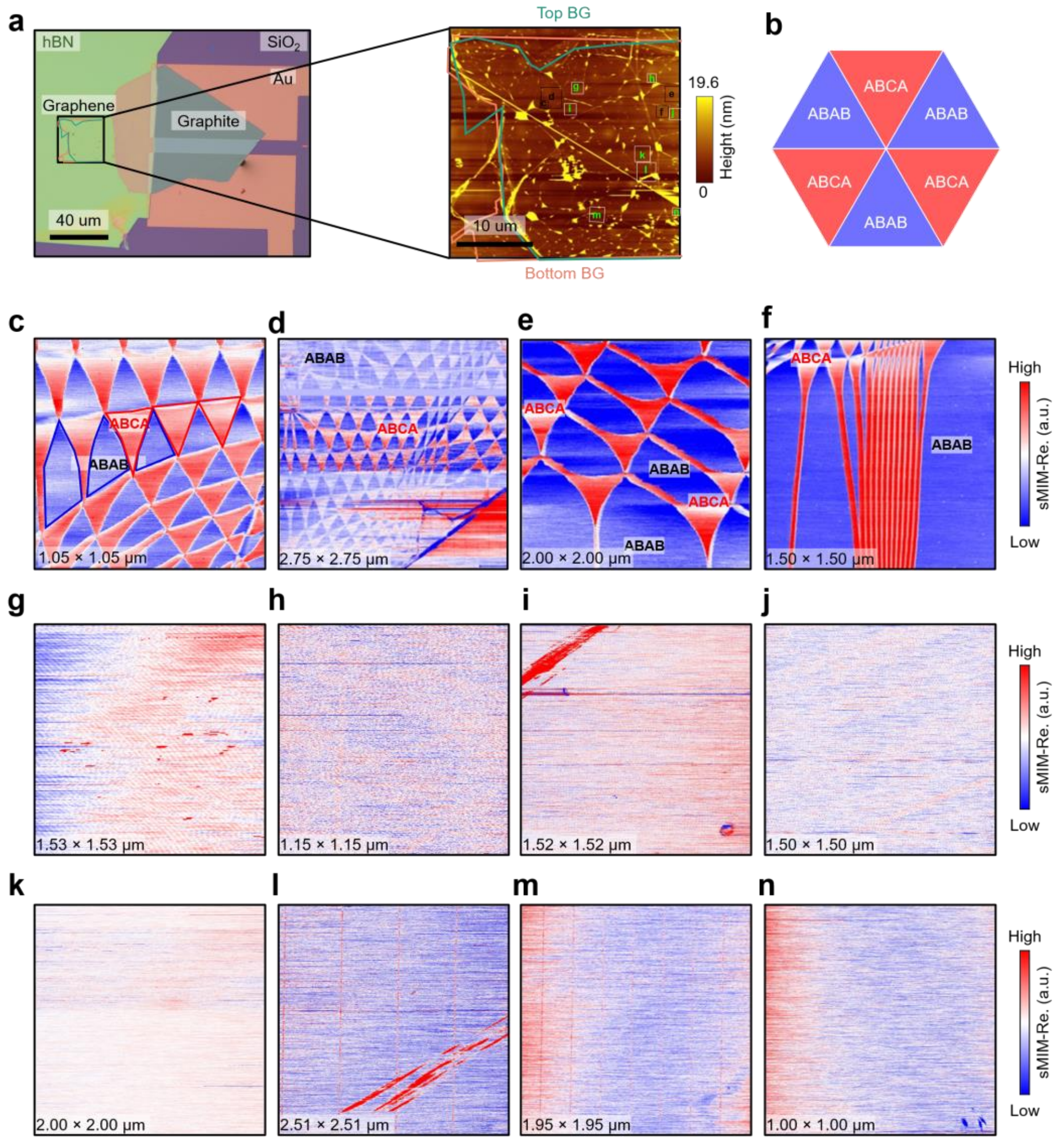

Extended Data Fig. 3 | Optical photo and moiré superlattice of TDBG device. a, Optical photo of device structure (left) and a magnified AFM topography (right) of device at RT, showing several wrinkles confining blocks. **b**, Cartoon of moiré superlattice domain structure of marginally TDBG. **c-f**, Moiré superlattice of TDBG by sMIM-Re mapping at RT. The scan region is 1.05, 2.75, 2.00 and 1.50 μm, respectively. These regions show the convex ABAB and concave ABCA domains in marginally TDBG. The moiré superlattice size varies from ~100 nm to ~500 nm, while the quasi-hexagonal order and ABAB/ABCA configuration maintain. **g-n**, Additional sMIM-Re mappings acquired at RT across various widely separated regions, displaying a complete absence of moiré superlattices and confirming the dominance of uniform ABAB stacking. The moiré region in panel (f) is directly adjacent to the moiré-free region in panel (j), clearly capturing the structural evolution from a mixed ABCA/ABAB moiré state into a pure ABAB phase, with a similar spatial adjacency observed between the regions in panel (d) and panel (i).

3. To give a detailed explanation of coexistence of moiré and moiré-free region, we have revised the TDBG structure part in our main text (page 9, line 162): "Zooming into these individual blocks with room temperature sMIM-Re reveals varying

domain distributions, which points to slightly different local twist angles near zero. Specifically, some blocks exhibit a mixed domain state of ABAB and ABCA stacking with varying moiré superlattices ranging from 100 nm to 500 nm (Extended Data Fig. 3c–f), with the ABAB domains consistently exhibiting a convexly expanded morphology due to their enhanced stability and lower energy relative to the ABCA configuration. In contrast, extensive regions within other confined blocks completely lack moiré superlattices, having fully relaxed into a uniform ABAB stacking phase (Extended Data Fig. 3g–n). We therefore conclude that the ABAB stacking phase robustly dominates the overall device area, regardless of the local presence of a moiré superlattice."

4. We have revised the main text part discussing the signal contribution from ABAB domains (page 12, line236): "These results indicate that the Bernal configuration dominates the observations, likely because it covers a larger portion of the sample regardless of the local presence of moiré superlattices. Although ABCA-stacked domains are also present in a few blocks, their fraction appears too small to contribute appreciably to the measured signals, rendering their associated features undetectable in our experiment (Extended Data Fig. 4d–g)."

5. To provide a comprehensive view of our experimental constraints, we have expanded the Discussion section in the revised manuscript. The added paragraph clarifies the mechanical limitations of the current SCCM technique and explains why resolving distinct stacking domains *in situ* was not feasible (page 18, line 342): "A high-resolution SCCM spectrum on distinct stacking domains would provide valuable insights into the origin of the QHIFM states and its relation to the moiré superlattice. However, despite our best efforts, the mechanical noise level in the dry-dilution fridge environment prevented us from resolving the moiré superlattice *in situ*. To rule out ambiguity and confirm that our spectroscopic data originates from the dominant ABAB phase, we conduct extensive sMIM line scans across large areas (> 10 μm) spanning from the adjacent BG region deep into the TDBG region (Extended Data Fig. 10 b-d). These large-scale scans reveal the ubiquitous presence of the QHIFM state sequences throughout the entire TDBG region. While the signal amplitude and the exact gate voltage onset of these states exhibit spatial fluctuation due to charge disorder, the characteristic sequence of the QH states remains robustly intact everywhere (consistent with Fig. 2d). We do not observe any regions exhibiting the distinct spectrum expected for ABCA stacking. Furthermore, we perform high-resolution sMIM 2D mapping over a 3×3 μm region at various filling factors (Extended Data Fig. 10e, left). Although the mechanical noise prevented direct imaging of the moiré lattice, we observe subtle spatial patterns near certain QHIFM states (e.g. ν = -7 and +1), which might be the signature of the

underlying moiré superlattice. Importantly, the spectroscopic signature of sMIM line scan across this area reveals a clearly resolved and uniform QHIFM sequence (Extended Data Fig. 10e, right). Despite local density variations and possible moiré-induced textures, the fact that the characteristic QHIFM sequence is observed throughout the sample confirms that the signal is dominated by the robust ABAB phase."

# Question 1-5:

If they cannot, an alternative approach is to perform SCCM in naturally existing tetralayer graphene with either ABAB- or ABCA- stacking. It seems to me that, the LL crossing is just a property of multilayer graphene as discussed in ref. 41 (PRB, 83, 165443 (2011)), and doesn't reply on the formation of Moire domains created in TDBG. I also found no consideration of Moire domains in their theoretical calculation. Therefore, it is informative to compare SCCM results on ABCA-stacked and ABAB-stacked tetralayer graphene. By performing such experiments, one can directly assess the capability of SCCM to resolve LL crossing in energy spectrum. One may also gain insights to the discrepancy in current TDBG device, which could be related to the twisting-induced disorders.

# Reply:

We thank the reviewer for this insightful comment. We fully agree with the physical picture that the LL crossing behavior is an intrinsic property of multilayer graphene. Indeed, as the reviewer noted, our theoretical calculation does not include the moiré potential—precisely because the electronic properties of marginally twisted double bilayer graphene, once relaxed, are known to be independent of the moiré superlattice, as extensively documented in the literature.

In previous STM study on marginally TDBG, Kerelsky et al. [PNAS 118, e2017366118 (2021)] demonstrated that the system forms large, uniform commensurate domains by pushing the unnatural atomic stackings of the spatially evolving TDBG moiré to the domain walls and decoupling the ABCA and ABAB graphene from the effects of the superlattice potential. Although they use a marginally TDBG moiré to achieve the ABCA/ABAB graphene regions, they are no longer affected by the much larger moiré, allowing the study of ABCA/ABAB graphene without any superlattice potential affecting its low-energy electronic properties. This "moiréless" picture is further confirmed by a recent STM study by Liu et al. [Phys. Rev. Lett. 135, 156401 (2025)]. They verified that the electronic structure of the relaxed domains in marginally TDBG is the same as that of intrinsic tetralayer graphene, as detailed in their Supplemental Material Fig. S11 and Fig. S12. Our sample shows similar domain configuration and

twist angles. Therefore, treating the Hamiltonian as pristine tetralayer graphene is supported by the "moiréless" nature of these domains.

Moreover, our identification of the dominant ABAB phase is supported by a recent transport study on marginally TDBG by Brandon et al. [arXiv:2510.00220 (2025)]. They report a doping-induced sliding phase transition between the Bernal-dominant and Bernal-Rhombohedral mixed states. Their results confirm that the Bernal (ABAB) stacking is the energetically dominant ground state over large areas. While our spatially resolved measurements focus on the local conductivity and chemical potential rather than the global switching reported in their work, the observation of Bernal dominance is consistent across both studies. This aligns perfectly with our observation of ubiquitous QHIFM sequences and LL crossing behaviors throughout our sample.

While we fully recognize the scientific value of a direct control experiment, fabricating an open surface pristine ABCA graphene device is known technically challenging due to its metastable nature. The primary focus of this work is to establish SCCM as a powerful thermodynamic probe for QHIFM systems, using the BG and TDBG platform as a demonstration testbed. Based on these related literatures and our own data, we believe that our current measurements on the relaxed TDBG domains provide sufficient evidence to validate our conclusions, as these domains are established to be "moiréless" and dominated by the ABAB phase.

We again agree with the reviewer's suggestion on site-specific SCCM spectroscopy. Addressing the vibration limitations in dry-DR SCCM will enable the future in-situ investigation of distinct stacking domains and potentially the visualization of gate-driven domain sliding dynamics in arXiv:2510.00220 (2025).

# Revision:

1. To further explain the dominant ABAB phase, we have added a new paragraph in the Discussion section (page 18, line 360): "Moreover, our identification of the dominant ABAB phase is supported by a recent transport study on marginally TDBG[42]. They report a doping-induced sliding phase transition between the Bernal-dominant and Bernal-Rhombohedral mixed states. Their results confirm that the Bernal (ABAB) stacking is the energetically dominant ground state over large areas. While our SCCM measurements focus on the local conductivity and chemical potential rather than the global switching reported in their work, the observation of Bernal dominance is consistent across both studies. This aligns perfectly with our observation of ubiquitous QHIFM sequences and LL crossing behaviors throughout our sample.
2. To clarify the "moiréless" nature of TDBG, we have added a new paragraph in the Discussion section (page 18, line 367): "How would moiré potential affect the

QHIFM states in the ABAB domains? Previous study demonstrated marginally TDBG forms large, uniform commensurate domains by pushing the intermediate atomic stackings of the spatially evolving TDBG moiré to the domain walls and decoupling the ABCA and ABAB graphene from the effects of the superlattice potential[43]. Although a marginally TDBG moiré was used to achieve the ABCA/ABAB graphene regions, both domains are no longer affected by the much larger scale moiré, allowing the study of ABCA/ABAB graphene without any superlattice potential affecting its low-energy electronic properties. Such "moiréless" picture is further confirmed by a recent STM study[44], where the electronic structure of the relaxed domains in marginally TDBG is the same as that of intrinsic tetralayer graphene. Our sample shows similar domain configuration and twist angles. Therefore, treating the Hamiltonian as pristine tetralayer graphene is supported by the "moiréless" nature of these domains."

The added references are listed below:

42. Brandon, D. *et al.* Nonvolatile Switching of Magnetism via Gate-Induced Sliding in Tetralayer Graphene. Preprint at https://doi.org/10.48550/arXiv.2510.00220 (2025).

43. Kerelsky, A. *et al.* Moiréless correlations in ABCA graphene. *Proc. Natl. Acad. Sci.* **118**, (2021).

44. Liu, Y. *et al.* Electronic correlations in rhombohedral graphene at atomic scale. *Phys. Rev. Lett.* **135**, 156401 (2025).

# Question 1-6:

3)An important observation is the formation of symmetry-broken QHIFM states in TDBG, which are absent in neighboring BG area. Such QHIFM states also shows strong electron-hole asymmetry. The emergence of QHIFM states has to do with various spin or valley symmetry breaking process, which is apparently connected to the formation of Moire domains in TDBG device. The current experiment doesn't reveal much information about the microscopic origins of such symmetry breaking process, such as its relation to the Moire domain structure.

# Reply:

We thank the reviewer for highlighting the contrast between TDBG and BG regions. We attribute the emergence of QHIFM states in TDBG and their absence in the neighboring BG to the intrinsic properties of the ABAB-stacked tetralayer system—namely its distinct LL structures and wavefunction components—rather than to moiré superlattice effects or extrinsic structural factors. As discussed in the response to Question 1-4, the relaxed TDBG domains behave as "moiréless" tetralayer Bernal-

dominated graphene, which already rules out any significant contribution from the moiré potential. Beyond this structural consideration, extensive experimental results further support this interpretation, as detailed below.

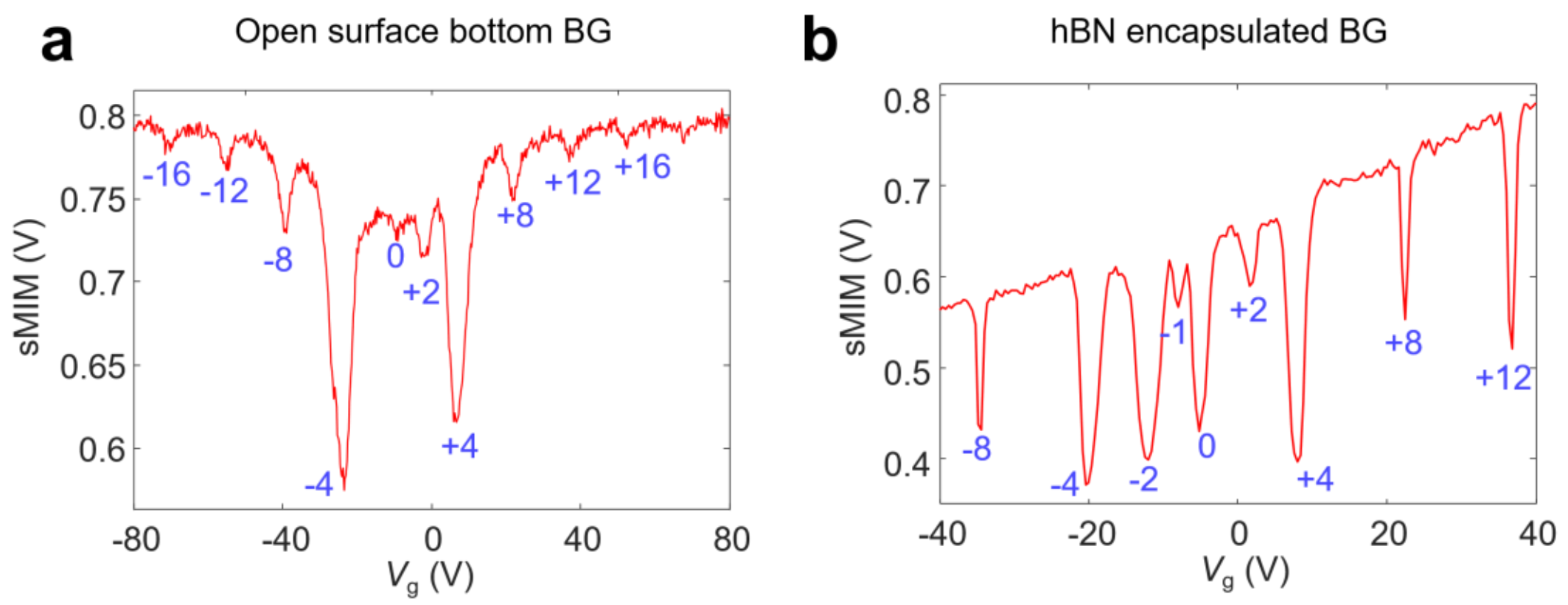

Figure R3. QHIFM states in different BG devices. a, Gate-dependent sMIM point spectroscopy (B = 9 T and T = 100 mK) of QH states measured on the open-surface bottom BG region corresponding to Fig. 1c in the main text. **b**, Gate-dependent sMIM point spectroscopy (B = 9 T and T = 100 mK) of QH states on an hBN encapsulated BG reference device. Blue numbers denote the LL filling factors of BG.

The zeroth LL of BG possesses an 8-fold degeneracy (spin, valley, and orbital N = 0,1). In our BG data, we observe the lifting of the spin and valley degeneracy near the CNP, while the orbital degeneracy generally remains intact (Fig. 1c and g). In Fig. R3a, we performed additional sMIM point spectroscopy on the bottom BG region of the same TDBG device. Although the signal-to-noise ratio is slightly lower, it reproduces partial splitting at CNP but preserves 4-fold symmetry in higher LLs (N > 1). In Fig. R3b, we measured an independent hBN-encapsulated BG device. We consistently observe the same broken-symmetry QH states (with additional ν = -1 state) only near the CNP and no additional QHIFM states in higher LLs. Same results were also reproduced in gate-dependent line scans through the BG region in Fig. R2 b-d. The QHIFM states near CNP are also consistent with recent STM study [Ref. 33, Nat. Phys. 21, 716–723 (2025)] on open surface BG. Therefore, these results prove the dominance and robustness of Coulomb interaction in N = 0 and 1 LLs. As for the higher LLs, we note the top BG shows a ν = -6 state (Fig. 1c and Fig 2.d) while other datasets show only ν = 4N QH sequences. The difference between bottom and top BG indicates that charge disorders could broaden the LLs and change the QH ground states. However, this sample-dependent variation stands in stark contrast to the intrinsic and robust QHIFM spectrum observed universally across the TDBG region.

As shown in Ref 41 [Phys. Rev. B 83, 165443 (2011)], the presence of next-nearest interlayer hopping γ_2 and γ_5 in tetralayer graphene causes an intrinsic single-particle splitting of the two bilayer-like subbands and their respective N = 0 and 1 orbitals. Also,

such orbital splitting scales with the magnetic field. This means that unlike BG, which starts from a highly degenerate manifold, the tetralayer LL spectrum is already split into a dense set of sub-bands at the single-particle level. As shown in Fig. 4d, these single-particle splitting induced low-energy LL subbands possess prominent lowest LL φ_0 and/or φ_1 orbital components, including B_{3-}, B_{2-}, b_{2-}, B_0, B_1, b_0, B_{2+}, b_1 and B_{3+}. These wavefunction structure significantly enhance the electron-electron interactions in multiple LL subbands, thereby accounting for the observed QHIFM sequences. Similar results were also reported by a previous transport work by Shi et al [Phys. Rev. Lett. 120, 096802]. Their transport measurement on ABAB graphene reveals all integer QH states at filling factor $-8 < v < 8$ (See Fig. S3 in Phys. Rev. Lett. 120, 096802). The difference might come from the open surface and enhanced interaction in our sample and fixed displacement field at D = 0 V/nm in their experiment. Despite these differences, both experiments show ABAB graphene possesses a group of highly interaction-dominated LL subbands.

We also note that Shi et al. observed $v = +5, +6, +7$ states, whereas our data resolves only even filling factors for $v > 4$ on the electron side (e.g., $v = +6, +10$). The absence of odd v states in the electron side results in significant electron-hole asymmetry in our measurement as noted by the reviewer. As shown by multiple sMIM gate-dependent line scans (Fig. 2d, Extended Data Fig. 4g, and Fig. R2b-e), the specific electron-hole asymmetry and QHIFM sequence persist robustly across different sample regions, despite the varying density and type of local charge disorder in these scans. This persistence suggests that the asymmetry is likely driven by intrinsic interaction mechanisms rather than random local potential fluctuations, although a definitive conclusion needs further theoretical and experimental investigation.

# Revision:

1. A new sentence has been added on page 16 line 324: " As shown by the sMIM gate-dependent line scans (Fig. 2d, Extended Data Fig. 4g and Extended Data Fig. 10), the specific electron-hole asymmetry and QHIFM sequence persist robustly across different sample regions, despite the varying density and type of local charge disorder in these scans. This persistence suggests that the asymmetry is likely driven by intrinsic interaction mechanisms rather than random local potential fluctuations, although a definitive conclusion needs further theoretical and experimental investigation."
2. To explain the difference of QHIFM states in BG and TDBG, a new paragraph is added in page 16 line 330: "Our measurements also clearly reveal distinct behaviors of the QHIFM states in the BG and TDBG regions. The zeroth LL of BG possesses an 8-fold degeneracy (spin, valley, and orbital $N = 0,1$). In our BG data, we observe

the lifting of the spin and valley degeneracy near the CNP, while the orbital degeneracy generally remains intact (Fig. 1c and g). In contrast, remote interlayer hopping in tetralayer graphene break this orbital degeneracy[41], creating a dense manifold of sub-bands at the single-particle level. As shown in Fig. 4d, these splitting-induced, multi-component LL subbands retain prominent nonrelativistic zeroth LL φ_0 and/or first LL φ_1 orbital components, including B_{3-}, B_{2-}, b_{2-}, B_0, B_1, b_0, B_{2+}, b_1 and B_{3+} branches. Ultimately, this single-particle splitting, combined with the multi-component nature and wavefunction structures of the sub-bands, accounts for the rich QHIFM sequences uniquely observed in TDBG."

Question 1-7:

On the other hand, the authors try to argue the connection of such QHIFM states to the LL crossing, which is not very convincing for me. As shown in Extended Data Fig. 8d, such QHIFM states (v=5,6,7) exist before and after the LL crossing. The event of LL crossing only shifts the position of QHIFM states, but is not its origin.

Reply:

We fully agree with the reviewer that such QHIFM states exist before and after the LL crossing. The formation of QHIFM states is driven by the strong Coulomb interactions intrinsic to the tetralayer graphene system. As discussed in Response 5, the "localized" nature of the φ_0 and φ_1 orbital components make the interaction energy dominant, driving the formation of QHIFM states before and after the LL crossing (as shown Extended Data Fig. 8d). We claim that the re-emergence behaviors are caused by LL crossing, which modifies the position/sequence of the QHIFM states, thereby resulting in the QHIFM phase diagram. This physical picture, where LL crossings reorganize the hierarchy of interaction-driven QHIFM states, is also consistent with previous transport work on ABA-stacked trilayer graphene [Ref 6, Nat. Commun. 8, 14518 (2017)], where interaction-enhanced gaps persist across complex crossing patterns.

Revision:

1. We have added a clarification in the main text to avoid ambiguity and further explain the effect of LL crossing on QHIFM phase diagram (page 16, line 304): "Consequently, the enhanced Coulomb interactions in b_0 give rise to QHIFM states at ν = +5, +6, and +7 below 5 T (Extended Data Fig. 8d), which subsequently transition to ν = +1, +2, and +3 above 6 T following the LL crossing (Fig. 4c, right). Crucially, the emergence of these QHIFM states is fundamentally driven by the strong intrinsic Coulomb interactions within the LL subbands with compact wavefunctions, a mechanism that persist robustly both before and after the LL

crossing. While the LL crossing defines the phase transition point, the specific ground state sequence is determined by the interaction-driven selection among these competing QHIFM states as well as single-particle effects such as the Zeeman energy and electric-field driven valley splitting. Therefore, our SCCM results clarify that the LL crossing acts as a modulator that reorganizes the sequence of the QHIFM states and shapes the QHIFM phase diagram, rather than serving as the microscopic origin of these states."

# Question 1-8:

4)The role of TDBG for their experimental observations is vaguely discussed. Apparently, such TDBG creates complex structural domain networks and other disordering factors (see Extended Data 3 and 4). Will such Moire superlattice be crucial for the LL crossing and resulting re-emergence of QH states? I doubt. The experiments I suggest in my 2nd comment can answer the question. Will this Moire superlattice in TDBG be crucial for the emergence of QHIFM states? It could be, but the discussion is very limited. The authors should properly discuss the role of TDBG (Moire superlattice) in their observation, so readers can understand the universality of the reported phenomena.

# Reply:

We thank the reviewer for this crucial comment regarding the universality of our observations. We again agree that clarifying the specific role of the TDBG platform and distinguishing between intrinsic ABAB physics and moiré-induced effects is essential for the interpretation of our results. We have discussed this 'moiréless' effect in the reply to Question 1-3, 4 and 5.

# Revision:

1. We have expanded the Discussion section in the revised manuscript to detail the ubiquitous presence of the QHIFM states across the sample, the macroscopic dominance of the ABAB phase, and the 'moiréless' nature of the TDBG domains.

Please address all the concerns raised before this manuscript can be considered for the publication.

Response to reviewer #2

In this paper, J. Hu et al. measured many-body Landau Level states in the moire system of multilayer graphene using an SCCM technique that combines KPFM and sMIM. By implementing feedback at three independent frequencies with three lock-in amplifier setups, the authors simultaneously measured and controlled AFM, KPFM (chemical potential), and sMIM (local conductivity) signals. Through this approach, they extracted the energy gap and DOS of Landau levels in TDBG and BG systems, ultimately completing the phase diagram of QHIFM. Beyond the methodological improvements, this study extends previous scanning probe microscopy investigations - previously limited to conventional 2D Landau systems in mono/bilayer graphene - to moire quantum Hall systems. It is a noteworthy point that the change of DOS with respect to the magnetic field was shown to be consistent with the calculation from Koshino, et al. (2011), and the occurrence of Landau level crossing in a specific range was clearly explained.

Overall, this paper is well-written, and presents a generally smooth logical flow, with clear terminology. However, I believe the following points require revision or further clarification.

# Reply:

We thank the reviewer for the positive feedback on our manuscript. In the following, we reply points by points to the questions.

# Question 2-1:

In Fig 2b-c, the temperature indicated in the panel (100mK) is not consistent with the caption (300mK).

Correction required: [Line 202] ‘Fig. 3a’ ◊ ‘Fig. 3b’

# Reply:

We thank the reviewer for pointing out this discrepancy with their careful reading. The temperature stated in the caption (300 mK) is correct, and the "100 mK" label in the original figure panels was an error.

To clarify the experimental conditions: while our point spectroscopy measurements are typically performed at a base temperature of 100 mK, the large-area XY spatial mapping (as shown in Fig. 2b-c) inevitably introduces heating due to the movement of the scanner. This dynamic scanning process raises the effective electron/sample temperature to approximately 300 mK during the measurement. However, as shown by our temperature-dependent spectra in Fig. 3e, the QHIFM states exhibit no abrupt

changes in their characteristics between 100 mK and 300 mK, with only a gradual variation in signal intensity.

We have thoroughly checked all other temperature labels across the main text and supplementary figures to ensure accuracy.

# Revision:

1. We have corrected the temperature labels in the panels of Fig. 2b-c to 300 mK in the revised manuscript.
2. In the revised manuscript, the typo has been corrected (page 12, line 210): "The chemical potential measurements also reveal a Landau fan-like feature (Fig. 3b)"

# Question 2-2:

Some of the 2D data plots in the paper lack colorbar labels and units. Without color labels, it's difficult to intuitively understand what the plot represents at a glance, and without unit indicators, quantitative interpretation of the data becomes challenging. Even if arbitrary units are used, they should be stated clearly. (e.g. Fig. 1f-g, Fig. 3a-b)

# Reply:

We sincerely thank the reviewer for pointing out this oversight. We fully agree that explicit colorbar labels and units are essential for the intuitive and quantitative interpretation of the data.

In the revised manuscript, we have carefully calibrated our data and labeled the colorbars with their actual physical measurement units: Volts for the sMIM signals and meV for the KPFM chemical potential. We believe these upgrades significantly enhance the readability and the quantitative depth of our experimental results. We appreciate the reviewer's constructive advice, which helped us elevate the presentation quality of the manuscript.

# Revision:

Explicit colorbar labels with actual physical units (e.g., V, meV) have been added to all sMIM and KPFM data plots.

# Question 2-3:

The explanation of the 'B, b' band notation used in Koshino et al. (2011) is not detailed enough in the main text, leading to a lack of clarity. Referring to and explaining Fig. 4(d) inset earlier in the text would help clarify for readers.

Reply:

We fully agree with the reviewer that a clear physical explanation of the 'B' and 'b' band notation is critical for the readability of the manuscript. The underlying basis transformation is essential for understanding the multi-component nature of the LL states discussed later.

Following the reviewer's valuable suggestion, we have substantially expanded the introduction of the band model at its first appearance in the main text. We now explicitly explain that, following Koshino et al. (2011), the tetralayer Hamiltonian is unitarily transformed into two nearly decoupled effective bilayer graphene systems: a low-mass b sector and a high-mass B sector. We emphasize that these effective bilayers do not reside on specific physical layers; rather, they are defined on a new set of basis states constructed from the linear combinations of the original sublattices on the odd (layers 1 and 3) and even (layers 2 and 4) layers.

Furthermore, as suggested, we have brought forward the reference to the inset of Fig. 4d. This provides readers with an immediate visual representation of the transformed sublattice basis and the corresponding Landau level wavefunctions.

Revision:

1. We have added detailed description of the origin of the light and heavy BG band and revised the Fig. 3 cd part in the main text (page 12, line 218): "To understand this complex LL spectrum, we adopt the model introduced by previous theoretical work[41] (see "Methods" for details). In this model, the Hamiltonian of the ABAB tetralayer graphene is unitarily transformed and decomposed into two weakly coupled effective BG systems: a light-mass sector (denoted as the b band) and a heavy-mass sector (denoted as the B band). Crucially, these effective BG bands are defined on a new set of orthonormal basis states constructed from the linear combinations of the original sublattices residing on the odd and even layers. In the presence of magnetic field, these two effective BG bands (b and B) give rise to two distinct sets of BG-like LLs. According to equation (1), the cyclotron frequency ω_c is inversely proportional to m^*, therefore the light-mass band b exhibits a larger energy spacing between LLs than the heavy-mass band B. The different dispersive slopes of these two sets of subbands would naturally result in a rich pattern of LL crossings. As shown in Fig. 3d, the calculated LL energy spectrum captures the widespread intersection of these two sets of LLs, where each LL is denoted by its subband (B or b) and LL index n (n+ for electron like states and n- for hole like states and $b/B_{0/1}$ for the zeroth LLs).

   Guided by the calculation results, we further classify the complex experimental LLs energy spectrum into two sets of BG-like LLs based on their dispersion with

magnetic field and corresponding Chern numbers (Fig 3c). Remarkably, our experimental LL energy spectrum closely matches with the theoretical results, successfully reproducing major LL crossing points, such as the crossing between b_0 and B_{2+} at $\nu = +4$, and the crossing between b_{2-} and $B_{0/1}$ at $\nu = -8/-4$."

2. We have revised the single-particle LL wavefunction part to give a clear and detailed explanation of the multi-component nature of LL subbands (page 15, line 264): "Based on previous theoretical work[41], the light-mass b subband and heavy-mass B subband behave as effective BG systems. A defining feature of these effective bilayers is that their low-energy Hamiltonian contains off-diagonal terms proportional to the square of the kinetic momentum (π^2). In a perpendicular magnetic field, the momentum operators act as raising and lowering operators for the non-relativistic LL wavefunctions φ_n. Specifically, at K valley, the n-th LL wavefunction $\psi_n^{b/B}$ is mainly given by $\psi_n^{b/B} = (\varphi_{n-2}, \varphi_n)$ residing on the two effective basis, which are linear combinations of odd/even layer sublattices. For instance, the K-valley wavefunction of b_{2-} is composed of φ_2 at β_2 and β_4 sites, and φ_0 at $\alpha_1$and α_3 sites, and the K-valley wavefunction of B_1 is only φ_1 at β_2 and β_4 sites. Here α_i and β_i are sublattices in the ith layer (Fig. 4a). The single-particle LLs wavefunction components for all low-energy subbands are summarized in Fig. 4d. Consequently, the underlying orbital character of these single-particle wavefunctions determines the electron cyclotron orbital extent, thereby governing the strength of the exchange interactions."

# Question 2-4:

In Extended Data Figure 3 (a), the shape of the marked area in the optical photo does not match the shape of the AFM topography boundary. And the sizes of the marked area in the AFM topography do not match the sizes of (c)-(f).

# Reply:

We sincerely thank the reviewer for the careful examination. The mismatched boundaries and aspect ratios were due to inaccuracies in our original manual annotations.

# Revision:

We have carefully updated Extended Data Fig. 3. Specifically:

1. We have corrected the marked region in the optical photograph (panel a, left) to accurately reflect the true boundary and shape of the AFM topography.

2. We have precisely resized and repositioned the marked area within the AFM topography (panel a, right) so that its dimensions and aspect ratio perfectly match the actual scanning field of view presented in panels (c)-(f).
3. We have included new zoom-in scans as panels (g)-(n). These new panels show reference regions without moiré superlattice.

# Question 2-5:

In Extended Data Figure 7, it is not clear how the energy gap was extracted from the chemical potential curve. The energy gap appears to be taken from the difference between the extrapolated lines of the two ends at the center value, but this center value does not always correspond exactly to the desired (even) integer filling. The choice of the fitting range also appears to be inconsistent (absolute/relative). Stating error/adding error bar may help.

# Reply:

We sincerely thank the reviewer for this constructive suggestion. We completely agree that a rigorous criterion is essential for determining the gap center and estimating the associated uncertainties. Following the reviewer's valuable suggestion, we have revised the gap and its error extraction procedure:

1. First, the local chemical potential $\mu(\nu)$ plateaus on both sides of the transition are fitted with linear functions to account for the background density of states:
$$\mu_{L(\nu)} = a_L\nu + b_L, \mu_R(\nu) = a_R\nu + b_R$$
where μ_L and μ_R represent the extrapolated baselines from the left and right plateaus, respectively.
2. We assume a Gaussian distribution for the disorder-induced broadening of the Landau levels, the chemical potential step can be modeled by the Gaussian error function, defined as: $\mathrm{erf}(x) = \frac{2}{\sqrt{pi}}\int_0^x e^{-t^2}dt$, which is the integral of the Gaussian density of states. We fit the transition region as:
$$\mu_{fit}(\nu) = \mu_L(\nu) + [\mu_R(\nu) - \mu_L(\nu)] \cdot \left[1/2 + 1/2 \cdot \mathrm{erf}\big((\nu - \nu_c)/w\big)\right]$$
where ν_c is the gap center and w is the broadening width.
3. The energy gap Δ is then defined as the vertical separation between the two extrapolated baselines at the fitted center ν_c:
$$\Delta = \mu_R(\nu_c) - \mu_L(\nu_c)$$
To quantify the uncertainty arising from the finite width of the transition, we define error bounds based on the full width at half maximum (FWHM) of the Gaussian

broadening, where FWHM $\approx$ $1.665 \cdot w$. The uncertainty $\delta\Delta$ is calculated as the variation of the gap size within the FWHM interval:

$$\delta\Delta = \frac{1}{2}|\Delta(\nu_c + \mathrm{FWHM}/2) - \Delta(\nu_c - \mathrm{FWHM}/2)|$$

Utilizing this standardized procedure above, the gap size and its uncertainty are extracted, accounting for both the local linear background and the physical disorder broadening.

# Revision:

1. We have updated Extended Data Fig. 7 using this new protocol. The figure now explicitly displays the erf fitting curves, the determined gap center, the FWHM ranges (shaded areas), and the rigorously calculated error bars for every extracted gap.
2. We have completely updated the caption of Extended Data Fig. 7 to detail this standardized fitting and error analysis procedure: The energy gaps and their error bounds are determined through the following procedure. First, the local chemical potential plateaus on both sides of the transition are fitted with linear functions $\mu_L(\nu)$ and $\mu_R(\nu)$ (yellow and orange dashed line) to account for the background density of states[47]. Then, the chemical potential step in the transition region is fitted (dark grey solid line) by $\mu_{fit}(\nu) = \mu_L(\nu) + [\mu_R(\nu) - \mu_L(\nu)] \cdot [1/2 + 1/2 \cdot \mathrm{erf}((\nu - \nu_c)/w)]$, where erf is the Gaussian error function, ν_c is the gap center and w is the broadening width. Finally, the energy gap Δ is defined as the vertical separation between the two extrapolated baselines evaluated at ν_c: $\Delta = \mu_R(\nu_c) - \mu_L(\nu_c)$. To quantify the uncertainty arising from the finite width of the transition, we define error bounds based on the full width at half maximum (FWHM, grey shaded area) of the Gaussian broadening. The uncertainty $\delta\Delta$ is calculated as the variation of the gap size within the FWHM interval: $\delta\Delta = \frac{1}{2}|\Delta(\nu_c + \mathrm{FWHM}/2) - \Delta(\nu_c - \mathrm{FWHM}/2)|$.
3. We have also included the error bars in Fig. 3e by this method.

# Question 2-6:

And this is my personal opinions regarding color selection.

In most of the 2-dimensional data plots, binary colormaps such as 'bwr' were used. Unless the data has a clear polarity or represents an on/off state where binary colors are particularly meaningful, this type of binary colormap can hinder intuitive interpretation.

Selecting a colormap that the contrast changes monotonically, or more intuitively perceptual colormap would improve readability. (e.g., Fig. 3b, Ext Fig. 8)

# Reply:

We agree with the reviewer's point regarding data visualization. Applying diverging binary colormaps (such as 'bwr') to monotonically changing physical quantities can introduce artificial visual boundaries and hinder intuitive interpretation.

Following this highly constructive advice, we have systematically updated the color schemes for all 2D data plots throughout the manuscript and the Extended Data. We have replaced the diverging binary colormaps with perceptually uniform, monotonic colormaps (specifically, the 'magma' and 'viridis' color scales). We believe this adjustment prevents any misleading emphasis on intermediate values, significantly enhances readability, and allows for a much more intuitive and quantitative interpretation of our data.

# Revision:

All diverging binary colormaps in the 2D data plots have been completely replaced with perceptually uniform, monotonic colormaps ('magma' and 'viridis') throughout the revised manuscript and extended data.

# Question 2-7:

In Fig. 4c, the symbols used for the middle energy gap and local conductivity have the same color and shape as the peak markers on the left, although they do not share the same physical meaning. I suggest changing the color of these symbols.

# Reply:

We thank the reviewer for pointing this out. We completely agree that using distinct symbols and colors is essential to avoid any visual confusion and to clearly separate quantities with different physical meanings.

# Revision:

1. We have changed the color of the symbols in Fig. 4c middle to avoid any visual confusion.

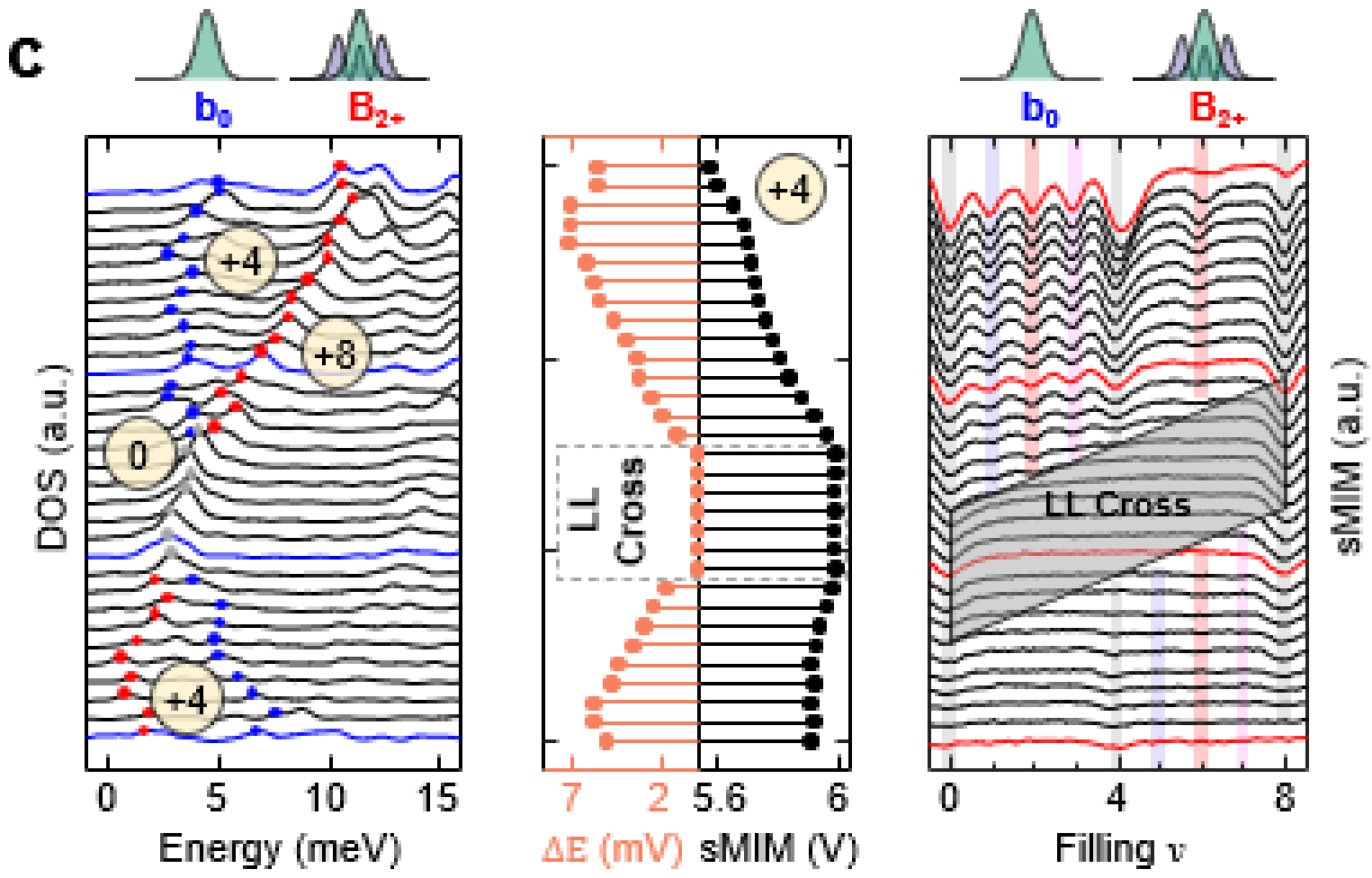

Fig. 4c. Left: waterfall plot of energy spectrum line profile at different B, shows the LL crossing between b0 and B2+. Middle: the evolution of LL energy splitting and conductivity of ν = +4 state. The consistence of energy splitting and conductivity confirms the LL crossing at ν = +4. Right: waterfall plot of sMIM as a function of filling factor ν at different B. A robust insulating state at ν = +1, +2, +3 manifests after LL crossing.

# Question 2-8:

These are personal questions and suggestions for future research that were not covered in this paper.

Have you attempted to verify whether the incompressible band generated by the isospin FM state is stronger and more uniform in the bulk rather than at the edge, and if it is generated uniformly at other edges as well?

When extending from the BG to the hBN region, do the local variations of the isospin states near the edge show consistency with the results of previous studies?

Will it be possible to more clearly confirm the continuity of the isospin FM state between BG and TDBG?

# Reply:

We thank the reviewer for these insightful comments and questions. The edge states of the QHIFM states and their evolution when approaching the edge are indeed central to understanding the topological nature of these systems. While some of these detailed spatial characterizations were beyond the primary scope of the current study, we have performed additional analysis and reference measurements to address these points.

In Fig. R3 (Extended data Fig. 10), we present three additional linecuts across the boundary between bilayer and tetralayer regions. These profiles demonstrate that the QHIFM states remain highly uniform across the bulk of the sample. While we do observe a relative attenuation of the QHIFM signal near the physical boundary, this

decrease is primarily attributable to extrinsic measurement artifacts rather than an intrinsic weakening of the isospin order. Specifically, variations in the tip-sample distance and spatial convolution effects from a finite tip radius likely integrate contributions from adjacent conductive chiral edge channels or the nearby bilayer region.

We agree with the reviewer that a careful examination on the continuity of the isospin FM state would provide valuable insights into their symmetry-broken nature. However, to definitively resolve the intrinsic behavior of these states at the edge, future studies utilizing high-resolution sMIM with optimized, ultra-sharp probes at closer tip-sample distance (e.g., via contact-mode sMIM) will be necessary. Furthermore, the intrinsic carrier density shifts between the BG and TDBG region hinders the examination of the isospin transition across the edge: we could not control the Chern number difference across the boundary. To analysis the isospin nature across the boundary, gate defined edge between different layer regions would be helpful to independently control the Chern number in each region so as to control the edge states and QHIFM states.

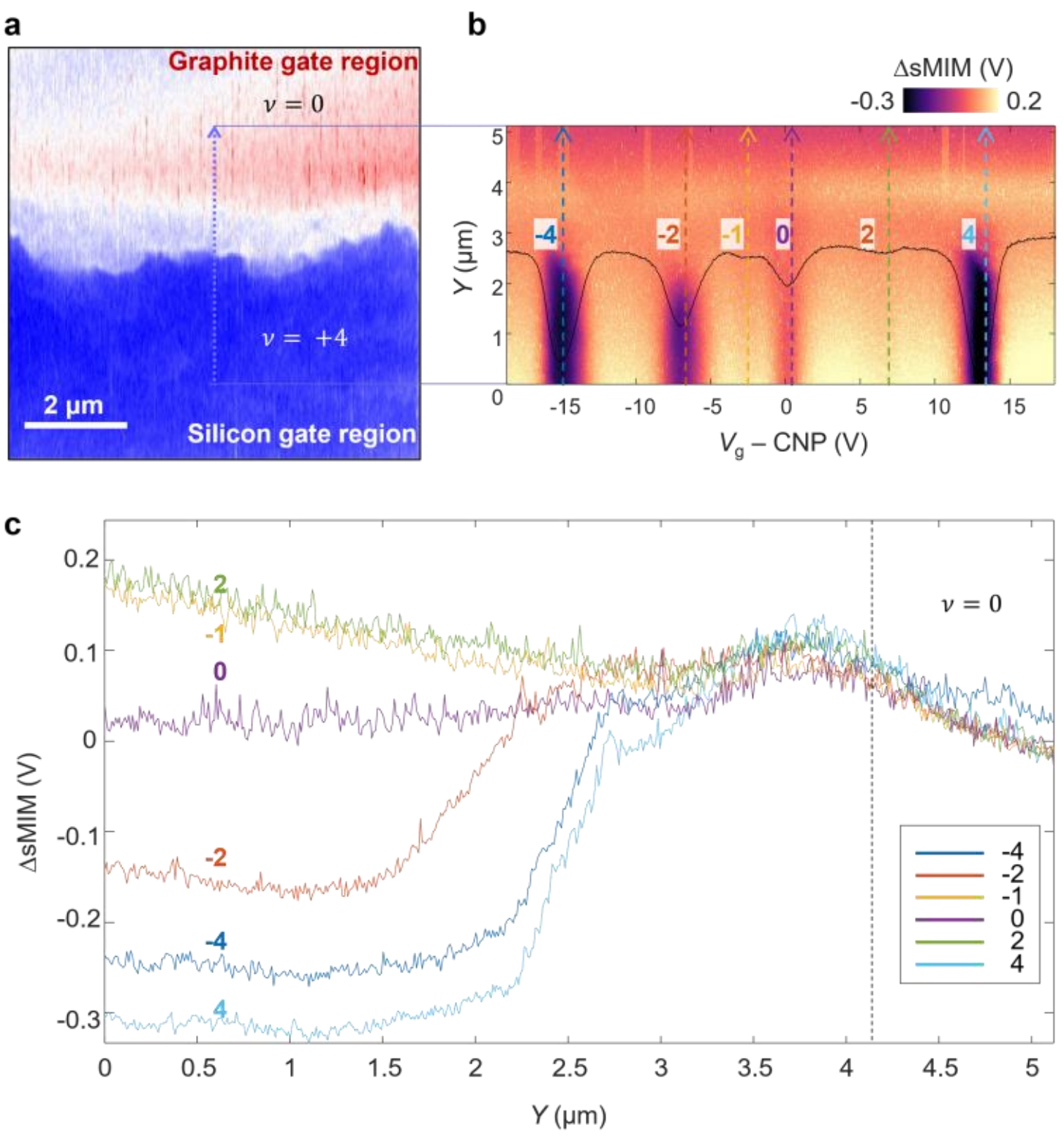

Figure R4. QHIFM states and its edge state across gate defined edge in another BG devices. a, sMIM-Im mapping of QH states of BG at $B = 9$ T. The graphite and silicon back are adjusted to form a gate-defined edge between $\nu = 0$ and $\nu = +4$ QH state. **b**, sMIM-Im silicon gate voltage dependent line scans at $B = 9$ T along the blue arrow indicated in **a**. Repeated line scans (y-axis) are performed while the silicon gate voltage is varied (x-axis). The graphite gate region is fixed at $\nu = 0$ QH state. The black line shows spatial averaged sMIM-Im in silicon gate region. **c**, sMIM-Im line profiles across the gate-defined edge for various QH states (Chern numbers indicated).

As mentioned by the reviewer, it is also very important to study the BG QHIFM states variation as approaching the hBN region. While we do not have direct spatial mapping data for the physical hBN edges on the primary sample, we have addressed this insightful inquiry by examining a reference bilayer graphene device featuring a gate-defined edge. In Fig. R4a, sMIM-Im mapping of the gate-defined QH states at 9 T shows distinct QH states $\nu = 0$ and +4 separated by conductive chiral edge states. We then fix the graphite gate region at $\nu = 0$ QH state and modulate the silicon gate region from $\nu = -4$ to +4 QH states. The silicon gate-dependent sMIM-Im linecut again shows QHIFM states at $\nu = -2, -1, 0$, and 2 (Fig. R4b). Fig. R4c details the sMIM-Im spatial profiles across this gate-defined boundary for various QH states. For $\nu = \pm 4$, we observe a highly incompressible bulk state (0-2.2 um) gradually evolving to chiral edge states at 3.8 um. Interestingly, there is a narrow incompressible stripe (2.75-3 um) with lower conductivity than $\nu = 0$ QH state. This incompressible stripe is also evident in Fig. R4a. In between 3.2-4 um, we observe a conductive chiral edge state region. For $\nu = -2$, similar behavior is also observed albeit without this evident incompressible stripe. Surprisingly, we observe an enhanced conductivity signal at the boundary even for $\nu = 0$ state. When both regions are tuned to $\nu = 0$ QH state, we would expect a uniform incompressible bulk state without chiral edge state separating them. Such result could be caused by some trivial effects like background capacitance changing when tip across from silicon gate region to graphite gate region, or convolution with a physical topographic height step. Nevertheless, the definitive identification of these sharp boundary transitions remains an exciting avenue for future high-resolution studies.

The methodological approach of this study is novel and has yielded results that could significantly influence future QHIFM research. With revisions and improvements to the points mentioned above, this paper will be suitable for publication in Nature Communications.

# Reply:

We sincerely thank the reviewer for the positive evaluation and recommendation.

Response to reviewer #3

The manuscript by Hu et al, presents a novel scanning technique that integrates scanning microwave impedance microscopy (sMIM) and Kelvin probe force microscopy (KFM) for probing correlated topological states in low-dimensional materials. The authors first establish the working principle of their techniques in Bernal bilayer graphene and observe the canonical generalized quantum hall ferromagnetic states and extract its Landau level spectrum. Next, they applied their technique to explore the quantum hall states in marginally twisted double bilayer graphene. The Landau fan obtained from the sMIM exhibits several missing sequences due to Landau level crossings. The authors were able to extract the detailed origin of these crossings thanks to the reconstructed many-body Landau level spectrum from the KFM mode. They further analyze these features and speculate on the properties of the Landau level wavefunctions that are most consistent with their data.

Overall, the data quality is very high, and the paper is well written. The technique introduced here will be a useful addition to the scanning probe community for probing the growing class of interesting van der Waals heterostructures. I recommend publication in Nature Communications provided that the authors address my following comments.

# Reply:

We sincerely thank the reviewer for their careful reading of our manuscript and the positive evaluation of our work. We are very much encouraged by the reviewer's recognition of our high data quality and the potential impact of our integrated sMIM and KPFM technique on the broader scanning probe community.

# Question 3-1:

1. Are the sMIM and KFM operations truly simultaneous?

# Reply:

We thank the reviewer for raising this important technical question. Yes, the sMIM and KPFM operations are truly simultaneous and are acquired in a single pass over the sample surface.

As detailed in Extended Data Fig. 1 and the Methods section of our manuscript, this simultaneous acquisition is achieved through the strict frequency separation of the excitation and readout signals. Crosstalk between the two SPM techniques is completely eliminated by employing distinct mechanical vibration eigenmodes of the cantilever and corresponding lock-in frequencies:

1. sMIM Operation (GHz regime): The microwave impedance is probed using a 3 GHz signal delivered to the tip apex. To eliminate drifting background, we demodulate the mechanical oscillation of the sMIM-Im signal at the cantilever's second eigenmode f_2=118 kHz.
2. KPFM Operation (kHz regime): Concurrently, for the Kelvin probe force microscopy, an AC voltage is applied to the tip at a frequency of f_2-f_1 = 100 kHz. The resulting electrostatic force is measured by demodulating the cantilever's mechanical response at f_1 = 18 kHz using a separate lock-in amplifier.

Because the sMIM electronics (operating at 3 GHz) and the KPFM/AFM feedback loops (operating at 18 kHz, 100 kHz, and 118 kHz) process signals that are separated by multiple orders of magnitude or assigned to distinct, non-overlapping eigenmodes, there is zero cross-talk between the measurement channels. This allows us to seamlessly apply all excitations to the same scanning tip and read out both the chemical potential and the local conductivity simultaneously in a constant-height, single-pass mode.

# Question 3-2:

2. Why there are more incompressible states near CNP in the TDBG region than in the BG region?

# Reply:

We thank the reviewer for highlighting this important physical distinction. The emergence of a higher number of incompressible states near the CNP in the TDBG region compared to the BG region is driven by a combination of intrinsic single-particle band splitting and electron-electron interactions.

The zeroth LL of BG possesses an 8-fold degeneracy (spin, valley, and orbital N = 0,1). In our BG data, we observe the lifting of the spin and valley degeneracy near the CNP, but the orbital degeneracy generally remains intact, resulting in fewer incompressible states (Fig. 1c and g). As shown in Fig. R3 and Fig. R4, additional reference BG devices consistently show similar broken-symmetry QH states near the CNP. In contrast, as established in Ref. 41 [Phys. Rev. B 83, 165443 (2011)], the presence of next-nearest interlayer hopping γ_2 and γ_5 in tetralayer graphene causes an intrinsic single-particle splitting of the two bilayer-like subbands and their respective N = 0 and 1 orbitals. This means that unlike BG, which starts from a highly degenerate manifold, the tetralayer LL spectrum is already split into a dense set of sub-bands at the single-particle level.

As shown in Fig. 4d, based on the single-particle splitting in tetralayer graphene, these low-energy LL subbands induced by single-particle splitting possess prominent lowest-LL φ_0 and/or φ_1 orbital components (e.g., B_{3-}, B_{2-}, b_{2-}, B_0, B_1, b_0, B_{2+}, b_1 and B_{3+}).

These wavefunctions with compact LL orbitals significantly enhance electron-electron interactions and give rise to observed QHIFM sequences.

Therefore, the combination of a denser single-particle spectrum and strongly enhanced correlation effects accounts for the rich sequences of QHIFM states observed universally across the TDBG region.

# Revision:

To explain the difference of QHIFM states in BG and TDBG, a new paragraph is added in page 16 line 330: "Our measurements also clearly reveal distinct behaviors of the QHIFM states in the BG and TDBG regions. The zeroth LL of BG possesses an 8-fold degeneracy (spin, valley, and orbital $N = 0,1$). In our BG data, we observe the lifting of the spin and valley degeneracy near the CNP, while the orbital degeneracy generally remains intact (Fig. 1c and g). In contrast, remote interlayer hopping in tetralayer graphene break this orbital degeneracy[41], creating a dense manifold of sub-bands at the single-particle level. As shown in Fig. 4d, these splitting-induced, multi-component LL subbands retain prominent nonrelativistic zeroth LL φ_0 and/or first LL φ_1 orbital components, including B_{3-}, B_{2-}, b_{2-}, B_0, B_1, b_0, B_{2+}, b_1 and B_{3+} branches. Ultimately, this single-particle splitting, combined with the multi-component nature and wavefunction structures of the sub-bands, accounts for the rich QHIFM sequences uniquely observed in TDBG."

# Question 3-3:

3. I suggest labeling the peaks in Fig. 2f with their Landau level index.

# Reply:

We thank the reviewer for this constructive suggestion, which significantly improves the clarity and readability of the data presentation.

In the revised manuscript, we have updated Fig. 2f to explicitly label the observed DOS peaks with their corresponding Landau level indices. Furthermore, to better correlate the data between panels, we have added horizontal dashed lines across Fig. 2e and Fig. 2f. These lines visually connect the labeled Landau level peaks in the DOS spectrum (panel f) directly to their corresponding chemical potential plateaus (panel e).

We believe this modification allows readers to much more intuitively and directly correlate the spectral features with the specific quantum Hall states discussed in the main text.

# Revision:

1. Figure 2f has been revised to include the explicit Landau level indices for all resolved peaks.
2. Horizontal dashed lines have been introduced to visually connect the DOS peaks in Fig. 2f with the chemical potential plateaus in Fig. 2e.

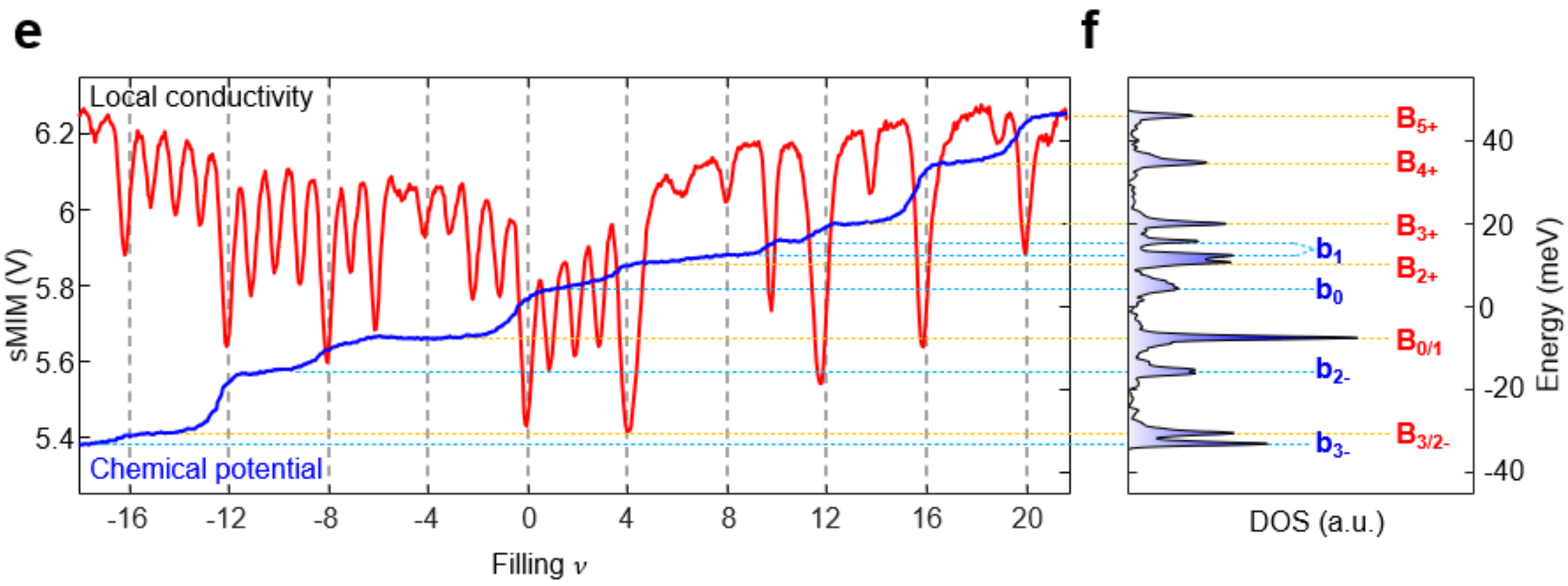

Fig. 2. e, The local conductivity (left axis) and chemical potential (right axis) of TDBG as a function of LL filling number ν at $T = 100$ mK and $B = 9$ T. All integer QH states at filling factor from +4 to -16 are clearly resolved in local conductivity, indicating fully broken-symmetry QHIFM states in TDBG. Chemical potential (blue curve) shows a sequence of plateaus, corresponding to LLs indicated in **f**. **f**, Thermodynamic DOS of TDBG extracted from the chemical potential curve in **e**. Each 4-fold LL is denoted by its subband (B or b) and LL index n (n+ for electron-like and n− for hole-like LLs), while the b1 LL is further split into two 2-fold subbands (see Extended Data Fig. 9 for details).
